# ANGPTL4 deficiency in haematopoietic cells promotes monocyte expansion and atherosclerosis progression

**DOI:** 10.1038/ncomms12313

**Published:** 2016-07-27

**Authors:** Binod Aryal, Noemi Rotllan, Elisa Araldi, Cristina M. Ramírez, Shun He, Benjamin G. Chousterman, Ashley M. Fenn, Amarylis Wanschel, Julio Madrigal-Matute, Nikhil Warrier, Jose L. Martín-Ventura, Filip K. Swirski, Yajaira Suárez, Carlos Fernández-Hernando

**Affiliations:** 1 Vascular Biology and Therapeutics Program, Yale University School of Medicine, New Haven, Connecticut 06520, USA; 2 Integrative Cell Signaling and Neurobiology of Metabolism Program, Section of Comparative Medicine and Department of Pathology, Yale University School of Medicine, New Haven, Connecticut 06520, USA; 3 Departments of Medicine and Cell Biology, Leon H. Charney Division of Cardiology and Cell Biology, New York University School of Medicine, New York, New York 10016, USA; 4 Center for Systems Biology, Massachusetts General Hospital and Harvard Medical School, Boston, Massachusetts 02114, USA; 5 Vascular Research Lab, IIS-Fundación Jimenez-Díaz, Universidad Autónoma de Madrid, Madrid 28040, Spain

## Abstract

Lipid accumulation in macrophages has profound effects on macrophage gene expression and contributes to the development of atherosclerosis. Here, we report that angiopoietin-like protein 4 (ANGPTL4) is the most highly upregulated gene in foamy macrophages and it's absence in haematopoietic cells results in larger atherosclerotic plaques, characterized by bigger necrotic core areas and increased macrophage apoptosis. Furthermore, hyperlipidemic mice deficient in haematopoietic ANGPTL4 have higher blood leukocyte counts, which is associated with an increase in the common myeloid progenitor (CMP) population. ANGPTL4-deficient CMPs have higher lipid raft content, are more proliferative and less apoptotic compared with the wild-type (WT) CMPs. Finally, we observe that ANGPTL4 deficiency in macrophages promotes foam cell formation by enhancing CD36 expression and reducing ABCA1 localization in the cell surface. Altogether, these findings demonstrate that haematopoietic ANGPTL4 deficiency increases atherogenesis through regulating myeloid progenitor cell expansion and differentiation, foam cell formation and vascular inflammation.

## Abstract (web summary)

Angiopoietin-like 4 protein (ANGPTL4) is a regulator of lipoprotein metabolism whose role in atherosclerosis has been controversial. Here the authors show that ANGPTL4 deficiency in haematopoietic cells increases atherogenesis by promoting myeloid progenitor cell expansion and differentiation, foam cell formation and vascular inflammation.

## Graphical abstract

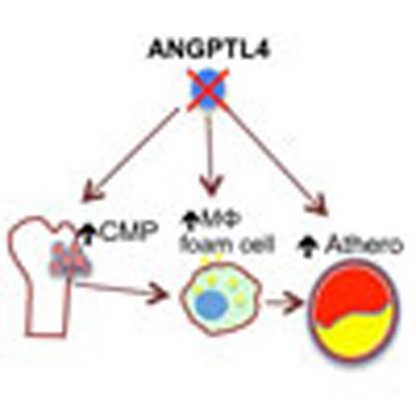

During the early stages of atherosclerosis, modified lipoproteins, primarily oxidized low density lipoproteins (Ox-LDL) accumulate in the intima, and activate endothelial and smooth muscle cells, recruit circulating monocytes into the sub-endothelial layer. Here, monocytes differentiate into macrophages, scavenge Ox-LDL, accumulate neutral lipids and transform into foam cells^1^,^2^. Foam cell formation is a protective mechanism whereby the vessel wall rids itself of potentially harmful lipids. However, accumulation of large numbers of foam cells in the arterial wall leads to the generation of atherosclerotic plaques^1^. Furthermore, both macrophages and foam cells play a key role in mediating inflammatory response in athero-plaques. Apart from foam cells, the monocyte count in blood circulation independently predicts risk for coronary artery disease after adjustment for conventional risk factors^3^. Monocytosis and neutrophilia have been observed in animal models of atherosclerosis including pigs and rabbits, and seem to contribute to atherogenesis^4^,^5^. Previous studies have demonstrated that hyperlipidemia-induced leukocytosis in different mouse models including *Abca1*^*−/−*^ or *Abcg1*^*−/−*^ and *Apoe*^*−/−*^ mice is associated with the expansion and proliferation of haematopoietic stem and multipotential progenitor cells (HSPCs) in the bone marrow (BM)^6^,^7^,^8^. Recent studies have shown that a family of proteins called angiopoietin-like proteins (ANGPTLs), particularly ANGPTL2 and ANGPTL5, are known to stimulate the expansion of haematopoietic stem cells *ex vivo*^9^,^10^,^11^. In addition, ANGPTL4 has been shown maintains *in vivo* repopulation capacity of CD34+ human cord blood cells^12^.

ANGPTL4 is a multifunctional protein that regulates many metabolic and non-metabolic processes through its distinct N-terminal and C-terminal domains^13^,^14^,^15^,^16^,^17^. Particularly, ANGPTL4 is a strong inhibitor of lipoprotein lipase (LPL), an enzyme that catalyses the hydrolysis of triglycerides (TG) from very LDL (VLDL) and chylomicrons, and regulates the uptake of circulating lipids into tissues^18^,^19^. As a result, overexpression of ANGPTL4 in mice leads to hypertriglyceridemia, whereas deficiency leads to lowering of circulating lipids^20^. Interestingly, human studies have shown that a common sequence variant near the *ANGPTL4* gene is associated with decreased plasma TGs and increased high-density lipoprotein cholesterol (HDL-C) levels, and ANGPTL4 expression is positively associated with metabolic parameters including levels of insulin, fatty acids and leptin^21^. Although decreased lipid content is generally atheroprotective, E40K, a loss of function variant of *ANGPTL4*, was associated with increased coronary heart disease risk despite being associated with an atheroprotective lipid profile, suggesting that ANGPTL4 influences parameters beyond lipid levels^22^.

ANGPTL4 is highly expressed in adipose tissues, placenta, liver, kidney and heart in humans^23^,^24^. It's expression is regulated by fasting, PPARs, glucocorticoids, and hypoxia in a tissue-specific manner^23^,^24^,^25^,^26^. ANGPTL4 is also highly abundant in macrophages and it is induced by dietary fatty acids^27^. *Angptl4*^*−/−*^ mice develop severe inflammation and accumulate foam cells in the mesenteric lymph nodes when fed a diet high in saturated fat^27^. This suggests that ANGPTL4 is a critical regulator of macrophage functions. Moreover, studies from overexpression or depletion of LPL in macrophages demonstrate that LPL promotes the binding and uptake of modified LDLs by macrophages and thus enhances foam cell formation^28^,^29^. ANGPTL4 can be expected to inhibit and reverse LPL-mediated effects in macrophages and atherosclerosis. However, there have been no studies addressing the direct role of macrophage ANGPTL4 during atherogenesis. Studies using global knockout or transgenic overexpression mouse models suggest both pro- and anti-atherogenic roles of ANGPTL4 (refs 30, 31). These confounding observations could have resulted from diverse roles of ANGPTL4 in regulating multiple metabolic parameters and inflammation, which could influence the progression of atherosclerosis.

In the present study, we demonstrate that haematopoietic-specific ANGPTL4 plays a critical role in the progression of atherosclerosis. We show that haematopoietic ANGPTL4 deficiency in *Ldlr*^*−/−*^ mice results in accelerated atherosclerosis characterized by bigger lesions, enhanced lipid accumulation, vascular inflammation and increased leukocytes in circulation. In doing so, we uncover a novel role of ANGPTL4 in the regulation of common myeloid progenitor (CMP) expansion and its subsequent differentiation into monocytes and neutrophils. We also provide additional mechanisms showing that ANGPTL4-mediated suppression of foam cell formation is a multifactorial process, involving increased lipoprotein influx and decreased cholesterol efflux from macrophages.

## Results

### ANGPTL4 is expressed in macrophages in atherosclerotic plaques

**Figure 1:**
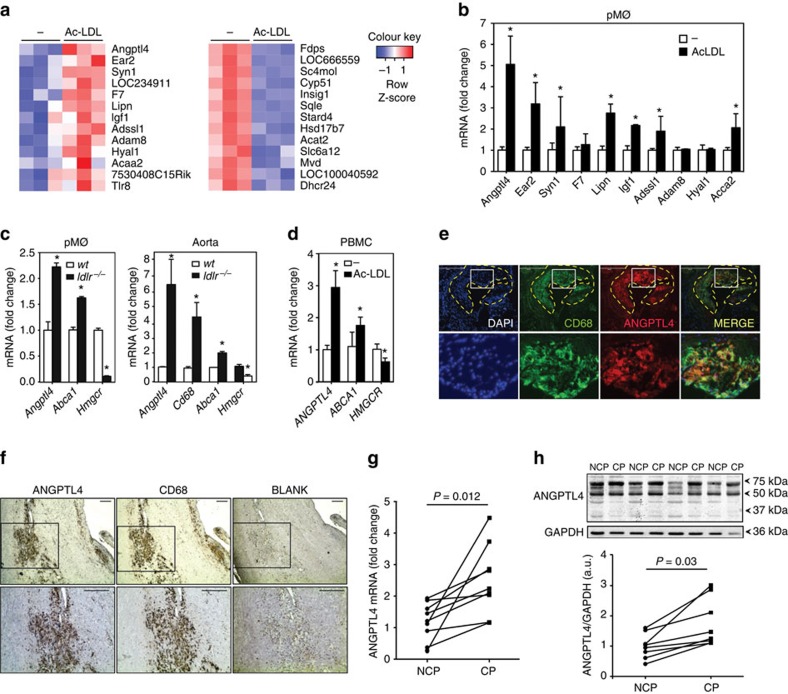
ANGPTL4 is expressed in macrophages accumulated in atherosclerotic plaques. (**a**) Heat map representation of gene expression from microarray data comparing mouse peritoneal macrophages incubated with or without Ac-LDL (120 μg ml^−1^) for 24 h. Left panel shows upregulated genes (*P*<0.05, unpaired *t*-test; fold change≥1.4) and right panel shows downregulated genes (*P*<0.05, unpaired *t*-test; fold change≥2.08) upon Ac-LDL treatment compared with non-treated cells. Samples were analysed in triplicate. (**b**) qRT-PCR validation of selected genes upregulated in the microarray in mouse peritoneal macrophages treated with or without Ac-LDL for 24 h. qRT-PCR analysis of *Angptl4* expression levels in macrophages (**c**, left) and whole aorta (**c**, right) from WT and *Ldlr*^*−/−*^ mice fed a WD (left) (*n*=4 per group), and in human peripheral blood mononuclear cells (**d**) treated with or without Ac-LDL (120 μg ml^−1^) for 24 h. *Abca1* and *Hmgcr* genes were used as control genes for cholesterol loading and *Cd68* was used as a marker for macrophages. All data represent the mean±s.e.m. and are representative of three experiments in duplicate; *P≤0.05 compared with untreated macrophages (**b**,**d**) and WT mice (**c**) by unpaired *t*-test. (**e**) Immunostaining of ANGPTL4 (red) and macrophage marker CD68 (green) and their co-localization in atherosclerotic plaques of *Ldlr*^*−/−*^ mice fed a WD. Scale bar, 400 μm. (**f**) Immunohistochemistry staining of ANGPTL4 and CD68 in human atherosclerotic plaques. Scale bar, 200 μm. (**g**) Comparison of *ANGPTL4* mRNA expression in NCP and corresponding culprit (CP) human atherosclerotic plaques (*n*=9). (**h**) Representative western blot showing comparison of ANGPTL4 expression in NCP and corresponding CP. Lower panel shows densitometry analysis for the 50 kDa bands of the western blots (*n*=7). *P≤0.05 compared with NCP by unpaired *t*-test; a.u. arbitrary units. Full scans of westerns blots are provided in Supplementary Fig. 7.

We initially aimed to identify genes that are modulated in macrophage-derived foam cells. To this end, we loaded thioglycollate-elicited mouse peritoneal macrophages with acetylated LDL (Ac-LDL), a synthetically modified form of native LDL to maximize cholesterol loading. Genes regulated at the mRNA level by cholesterol loading were then determined using an Affymetrix expression array (Supplementary Table 1). In addition to genes that have previously been associated with cholesterol metabolism, including *Cyp51*, *Dhcr24* and *Sqle* (Fig. 1a), we found a number of novel genes upregulated in macrophages incubated with Ac-LDL (fold change >1.4; *P*<0.05, unpaired *t*-test) (Fig. 1a). To confirm the results from the microarray study, a panel of 10 genes, that were identified as being highly upregulated upon cholesterol loading, were independently validated using quantitative real-time PCR (qRT-PCR) (Fig. 1b). Intriguingly, both microarray and qRT-PCR analysis revealed that *Angptl4* was the most highly and consistently upregulated gene in macrophage-derived foam cells (Fig. 1a,b).

We next measured the expression of ANGPTL4 in peritoneal macrophages isolated from *Ldlr*^*−/−*^ mice fed a Western-type diet (WD), which serves as an *in vivo* model of macrophage foam cells. As expected, macrophages isolated from *Ldlr*^*−/−*^ mice showed a marked up-regulation of *Abca1* and downregulation of *Hmgcr* compared with those from wild-type (WT) mice, indicating a massive accumulation of cholesterol (Fig. 1c). Importantly, *Angptl4* levels were significantly upregulated in macrophages and the whole aorta of *Ldlr*^*−/−*^ mice compared with WT mice (Fig. 1c). We also confirmed these results in primary human peripheral blood mononuclear cells (PBMC) treated with Ac-LDL (Fig. 1d). Notably, immunohistochemistry analysis of mouse aortic sinus plaques (Fig. 1e) and human carotid artery plaques (Fig. 1f) revealed a significant co-localization of ANGPTL4 and CD68, a macrophage marker, confirming that ANGPTL4 is highly expressed in macrophages in atherosclerotic plaques. Moreover, culprit plaques (CPs) from human carotid arteries (characterized by an advanced plaque area with massive infiltration of macrophages) showed significantly higher expression of ANGPTL4 at both the mRNA and protein levels compared with their adjacent non-complicated plaques (NCPs) (characterized by fibrotic areas with less inflammation) (Fig. 1g,h). In addition to the full-length ANGPTL4 (∼50 kDa) band, Western blot analysis showed stronger (∼75 kDa) and fainter (∼30 kD) bands, possibly representing oligomeric and individual truncated forms of ANGPTL4, as reported in previous studies^19^. Collectively, these data demonstrate that accumulation of lipids, which characterizes the formation of macrophage-derived foam cells, also results in enhanced expression of ANGPTL4 *in vitro* and *in vivo.*

Scavenger receptors, including CD36 and SR-A, mediate modified lipoprotein uptake in macrophages^32^,^33^. To determine whether Ac-LDL regulates *Angptl4* expression in a CD36-dependent manner, macrophages isolated from WT and *Cd36*^*−/−*^ mice were treated with Ac-LDL and *Angptl4* expression was compared. Surprisingly, there was no difference in the expression of *Angptl4*, indicating that CD36 is dispensable for this process (Supplementary Fig. 1a). Importantly, Ox-LDL, but not free cholesterol loading using cyclodextrin (Chol-CD), also induced *Angptl4* expression in macrophages, suggesting that additional species, such as fatty acids within Ac-LDL or Ox-LDL rather than cholesterol, might be involved in this induction (Supplementary Fig. 1b). Indeed, previous reports have demonstrated that members of a subfamily of the nuclear receptors, the PPARs, which are known to regulate *Angptl4* expression in several cell types^23^,^24^,^34^, are able to sense and interpret fatty acid signals derived from dietary lipids and pathogenic lipoproteins^35^. Consistent with these observations, Ac-LDL enhanced human *ANGPTL4* PPAR response element (PPRE) activity in Raw264.7 cells, indicating that Ac-LDL increased *Angptl4* expression via PPAR signalling (Supplementary Fig. 1c).

The presence of areas of low oxygen tension (hypoxia) is a common feature of atherosclerotic lesions^36^,^37^. *Angptl4* is a HIF-1α target gene and is known to be elevated under hypoxic conditions in a number of cell types^26^,^38^,^39^. To determine whether hypoxia stimulates *Angptl4* expression in macrophages as well, we exposed mouse peritoneal macrophages to hypoxic conditions using hypoxic chambers or CoCl_2_, a chemical inducer of HIF-1α. *Angptl4* expression was upregulated under both conditions, along with HIF-1α responsive genes *Glut1* and *Nos2* (Supplementary Fig.1d,e). Together, these findings demonstrate that *Angptl4* expression in macrophages is regulated by PPARs and hypoxia.

### Global ANGPTL4 deficiency attenuates atherosclerosis progression

**Figure 2:**
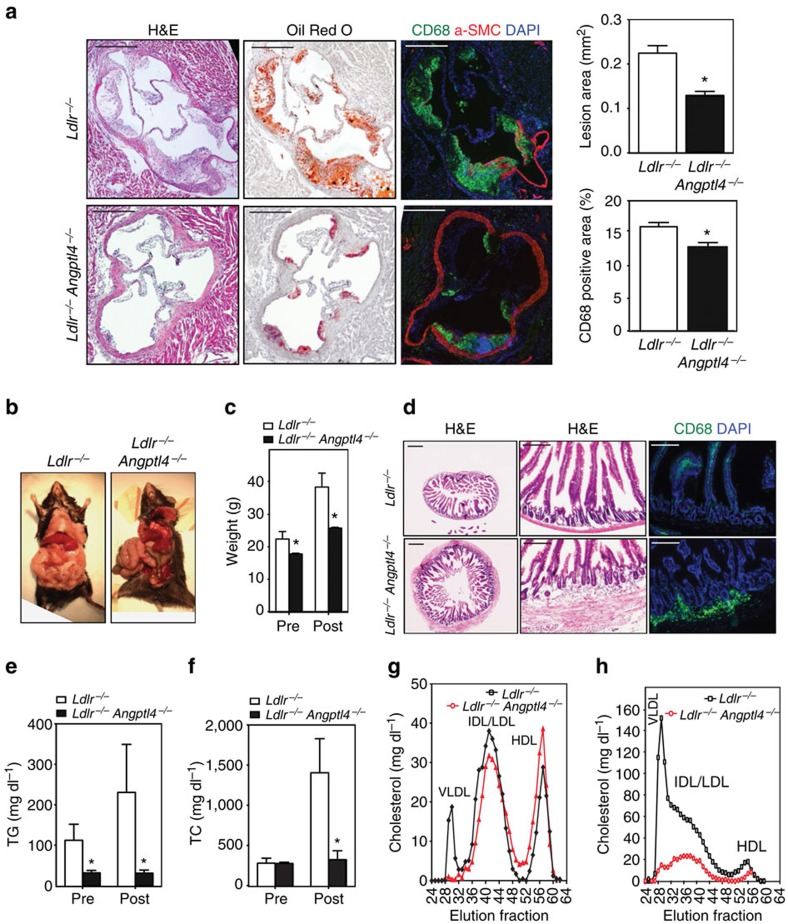
Absence of ANGPTL4 attenuates the progression of atherosclerosis. (**a**) Representative histological analysis of cross-sections of the aortic sinus from *Ldlr*^*−/−*^ and *Angptl4*^*−/−*^*Ldlr*^*−/−*^ mice stained with H&E, Oil Red O and CD68/SMC-actin/DAPI. Quantification of the lesion area and macrophage content is shown in the right panels (*n*=9 per group). Scale bars, 400 μm. (**b**) Representative pictures of whole body *Ldlr*^*−/−*^ and *Angptl4*^*−/−*^*Ldlr*^*−/−*^ mice on WD diet 12 weeks. (**c**) Body weight analysis of *Ldlr*^*−/−*^ and *Angptl4*^*−/−*^*Ldlr*^*−/−*^ mice before and after WD diet (*n*=9 per group). (**d**) Representative histological analyses of cross-section of the ileum from *Ldlr*^*−/−*^ and *Angptl4*^*−/−*^*Ldlr*^*−/−*^ mice stained with H&E and macrophage marker CD68. Scale bars, 400 μm. (**e**–**h**) Measurement of plasma TG (**e**), cholesterol (**f**) and lipoprotein profile from pooled plasma (**g**-**h**) of *Ldlr*^*−/−*^ and *Angptl4*^*−/−*^*Ldlr*^*−/−*^ mice before and after 12 weeks on WD diet (*n*=9 per group). All data are the mean±s.e.m.; *P<0.05 by comparison with data from *Ldlr*^*−/−*^ mice by unpaired *t*-test.

Previous studies have reported conflicting results concerning the role of ANGPTL4 in atherogenesis^31^,^40^. To dissect the importance of ANGPTL4 during the progression of atherosclerosis, we bred *Angptl4*^*−/−*^ mice with the athero-prone *Ldlr*^*−/−*^ mice. Then, *Ldlr*^*−/−*^ and *Angptl4*^*−/−*^ *Ldlr*^*−/−*^ mice were fed a WD for 12 weeks. Interestingly, we observed a marked reduction in atherosclerosis in *Angptl4*^*−/−*^*Ldlr*^*−/−*^ mice compared with *Ldlr*^*−/−*^ mice (Fig. 2a). Moreover, macrophage accumulation was also significantly reduced in ANGPTL4-deficient atherosclerotic lesions (Fig. 2a).

In contrast to the apparently beneficial effects seen in atherosclerotic plaques, *Angptl4*^*−/−*^ *Ldlr*^*−/−*^ mice exhibited a severe phenotype with peritonitis, depleted fat deposition in the gut, decreased body weight and severe gut inflammation (Fig. 2b–d), consistent with previous reports of *Angptl4*^*−/−*^ mice fed a high-fat diet^27^,^41^. Additionally, we observed decreased food intake and survival in these mice, with almost a third mice (4 out of 13 mice) dying before 12 weeks on a WD diet. Gut cross-section analysis revealed a massive infiltration of CD68-positive macrophages in the intestinal wall of *Angptl4*^*−/−*^*Ldlr*^*−/−*^ mice (Fig. 2d). Not surprisingly, given the role of ANGPTL4 in inhibiting LPL, plasma TG levels in *Angptl4*^*−/−*^*Ldlr*^*−/−*^ were drastically reduced compared with *Ldlr*^*−/−*^ mice (Fig. 2e). Moreover, total plasma cholesterol levels and VLDL particles were also markedly reduced (Fig. 2f–h). Overall, our results show that ANGPTL4 deficiency reduces atherosclerosis; however, the extreme phenotype observed in *Angptl4*^*−/−*^ *Ldlr*^*−/−*^ mice suggests that the reduced atherosclerosis may be a secondary effect of the detrimental changes caused by whole body depletion of ANGPTL4. These confounding observations indicate that the metabolic abnormalities observed with whole body modulation of ANGPTL4 make assessment of the role of ANGPTL4 in atherosclerosis development complicated and thus, tissue-specific ANGPTL4 modulation might mitigate these systemic effects in multiple organs and provide meaningful data.

### Haematopoietic ANGPTL4 deficiency promotes atherogenesis

**Figure 3:**
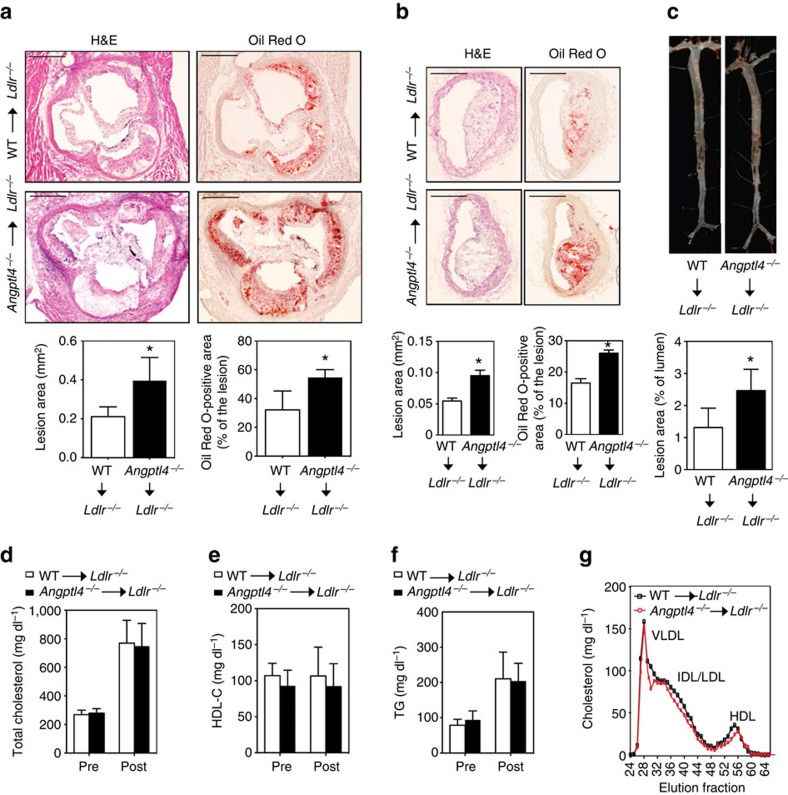
Haematopoietic ANGPTL4 deficiency enhances atherogenesis. (**a**,**b**) Representative histological analysis of cross-sections of the aortic sinus (**a**) and brachiocephalic arteries (**b**) isolated from male *Ldlr*^*−/−*^ chimeras with WT or *Angptl4*^*−/−*^ BM after 12 weeks of WD stained with H&E and Oil Red O. Quantification of lesion area and Oil Red O-positive lesion area is shown in the panel below respective figure (*n*=10 per group for aortic sinus and *n*=6 per group for brachiocephalic arteries). Scale bars, 400 μm. (**c**) Representative pictures from *en face* analysis of aortas from *Ldlr*^*−/−*^ chimeras with WT or *Angptl4*^*−/−*^ BM after 12 weeks on WD diet. Proportion of Oil Red O-positive area in the *en face* preparation is quantified in the lower panel (*n*=8 per group). Total cholesterol level (**d**), HDL cholesterol level (**e**) and TG level (**f**) in the blood plasma of *Ldlr*^*−/−*^ chimeras with WT or *Angptl4*^*−/−*^ BM before and after 12 weeks on WD (*n*=10 per group). (**g**) Lipoprotein profile from pooled plasma (*n*=5 each group) of *Ldlr*^*−/−*^ chimeras with WT or *Angptl4*^*−/−*^ BM after 12 weeks on WD diet. All data are the mean±s.e.m.; *P<0.05 by comparison with data from *Ldlr*^*−/−*^ chimeras with WT BM by unpaired *t*-test.

Given that ANGPTL4 is highly expressed in macrophage-derived foam cells, we sought to determine the role of macrophage-specific ANGPTL4 during atherogenesis. To this end, *Ldlr*^*−/−*^ mice were lethally irradiated and transplanted with the BM from *Angptl4*^*−/−*^ and WT donor mice. Reconstitution of the donor BM was confirmed by genotyping the blood (Supplementary Fig. 2). Then the mice were fed a WD for 12 weeks and atherogenesis was assessed. In contrast to the reduced atherosclerosis observed in germline ANGPTL4-deficient mice, *Ldlr*^*−/−*^mice transplanted with ANGPTL4-deficient BM had significantly larger atherosclerotic lesions compared with the ones with WT BM (Fig. 3a–c). Similar findings were observed in female *Ldlr*^*−/−*^ mice transplanted with *Angptl4*^*−/−*^ and WT BM (Supplementary Fig. 3a). Interestingly, the accelerated atherosclerosis observed in *Ldlr*^*−/−*^ mice reconstituted with ANGPTL4-deficient BM was not associated with difference in circulating plasma lipid levels (Fig. 3d–g and Supplementary Fig. 3c,d). Moreover, we did not find any differences in gut inflammation as assessed by CD68 staining and body weight, indicating that atherogenic effect observed in mice transplanted with *Angptl4*^*−/−*^ BM is independent of systemic inflammation (Supplementary Figs 3b and 4a,b).

**Figure 4:**
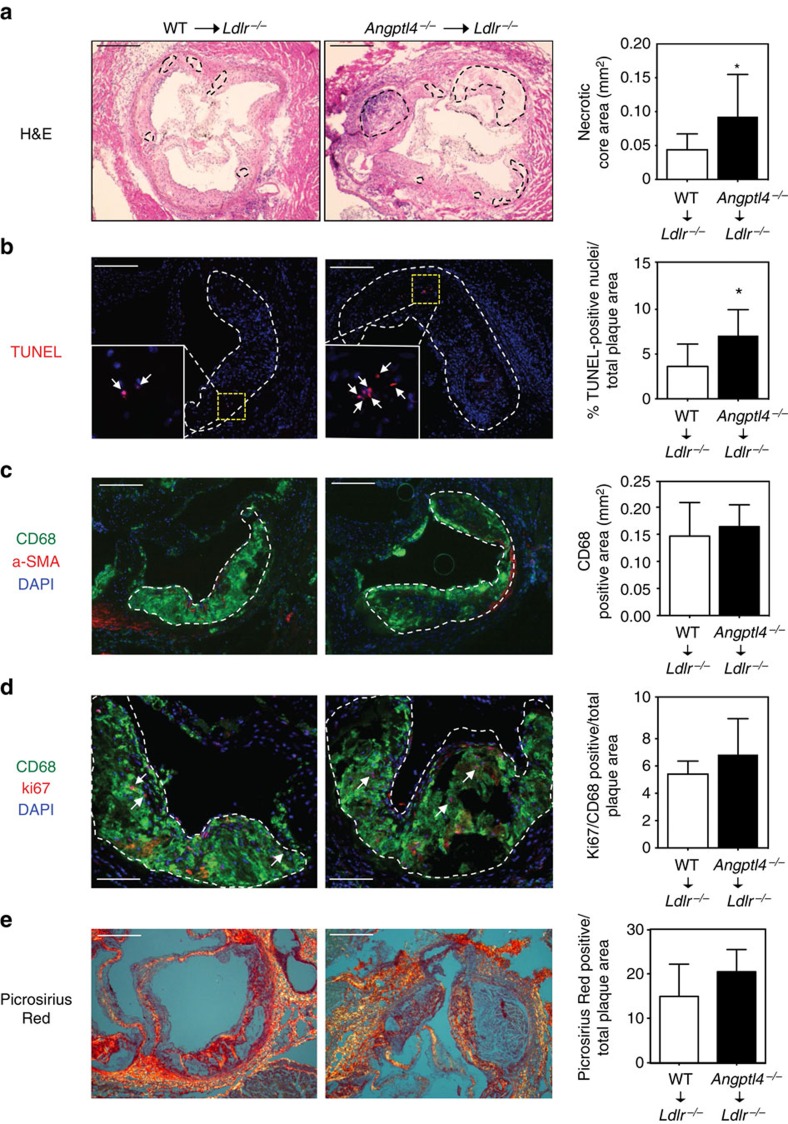
*Angptl4*^*−/−*^ BM recipients have more apoptosis in the atherosclerotic lesions. (**a**-**d**) Representative histological analyses of the cross-sections of the aortic sinus from *Ldlr*^*−/−*^ chimeras with WT or *Angptl4*^*−/−*^ BM after 12 weeks on WD stained with (**a**) H&E showing necrotic areas within dotted boundary (scale bars, 400 μm), (**b**) TUNEL; inset shows magnified TUNEL-positive area (scale bars, 200 μm), (**c**) CD68/α-SMA/DAPI (scale bars, 200 μm), (**d**) CD68/Ki67/DAPI (scale bars, 200 μm) and (**e**) Picrosirius Red staining (scale bars, 200 μm). Total necrotic area, TUNEL-positive nuclei, macrophage content, Ki67-positive macrophages and collagen content are quantified in the right panels. *N*=10 per group for **a**–**d** and *n*=6 per group for e. All data are the mean±s.e.m.; *P<0.05 by comparison with data from *Ldlr*^*−/−*^ chimeras with WT BM by unpaired *t*-test.

In human atherosclerotic vascular disease, plaque morphology is a more important predictor of plaque disruption and acute clinical events than plaque size^42^. In particular, the size of the necrotic core of advanced plaques is a key determinant of plaque vulnerability^43^. Analysis of lesions for acellular non-fibrotic areas revealed an increase in plaque necrosis in mice transplanted with *Angptl4*^*−/−*^ BM compared with the ones with WT BM (Fig. 4a). This increase could reflect a primary increase in macrophage apoptosis. Indeed, atherosclerotic plaques isolated from *Ldlr*^*−/−*^ mice transplanted with *Angptl4*^*−/−*^ BM showed a significant increase in TUNEL-positive cells (Fig. 4b). Moreover, cellular immunostaining showed that many of the TUNEL-positive nuclei were in macrophage-rich regions (Fig. 4b). Macrophage content in the atherosclerotic plaques was similar between groups despite larger lesions observed in *Ldlr*^*−/−*^ mice with ANGPTL4-deficient BM (Fig. 4c). Recent studies have shown that local proliferation dominates lesional accumulation of macrophages in advanced atherosclerosis^44^. Thus, we assessed if the absence of ANGPTL4 in macrophages influences macrophage proliferation *in vivo*. As shown in Fig. 4d, the number of Ki-67-positive cells was similar in atherosclerotic lesions in both groups of mice. Finally, we assessed the collagen content in the lesions, which is associated with plaque stability, using picrosirius staining. Despite a difference in the necrotic core area, collagen content was similar between groups of *Ldlr*^*−/−*^ mice transplanted with WT and *Angptl4*^*−/−*^ BM (Fig. 4e). This finding might be explained by the similar levels of macrophage accumulation in the plaques of both groups of mice, as macrophages are responsible for the modulation of collagen content by releasing metalloproteinases^45^. Taken together, these results indicate that ANGPTL4 deficiency in macrophages enhances the progression of atherosclerosis and increases macrophage apoptosis resulting in larger necrotic core in atherosclerotic lesions.

### Haematopoietic ANGPTL4 deficiency promotes leukocytosis

**Figure 5:**
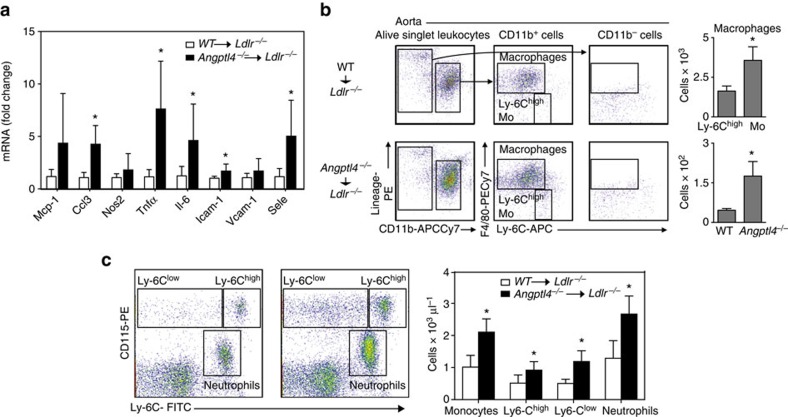
Haematopoietic ANGPTL4 deficiency causes vascular inflammation and leukocytosis. (**a**) mRNA expression of inflammatory genes in the whole aorta of *Ldlr*^*−/−*^ chimeras with WT or *Angptl4*^*−/−*^ BM after 12 weeks on WD (*n*=9 per group). (**b**) Dot plots showing gating schemes of macrophages and monocytes from whole aorta of *Ldlr*^*−/−*^ chimeras with WT or *Angptl4*^*−/−*^ BM after 12 weeks on WD. (right) The quantification of total number of macrophages and Ly-6C^high^ monocytes (*n*=6 per group). (**c**) Representative flow cytometry plots showing monocyte and neutrophil populations from *Ldlr*^*−/−*^ chimeras with WT or *Angptl4*^*−/−*^ BM after 12 weeks on WD. Total monocytes and neutrophils are quantified on the right panel by extrapolating the proportion of cells from flow cytometry to the total number of leukocytes per μl measured using Hemavet haematology analyser (*n*=10 per group). All data are the mean±s.e.m.; *P<0.05 by comparison with data from *Ldlr*^*−/−*^ chimeras with WT BM by unpaired *t*-test.

To appreciate the multiple mechanisms of atherogenesis in intact vessels, we analysed the quantitative expression patterns of inflammation in the vessel wall of *Ldlr*^*−/−*^ mice transplanted with WT or *Angptl4*^*−/−*^ BM after 12 weeks on WD by qRT-PCR. As expected by the increased atherogenesis observed in *Ldlr*^*−/−*^ mice reconstituted with *Angptl4*^*−/−*^ BM, the expression levels of several pro-inflammatory molecules including, *Tnfα*, *Ccl3* and *Il-6* and cell adhesion molecules *Icam-1* and *Sele* were significantly upregulated compared with *Ldlr*^*−/−*^ mice transplanted with WT BM (Fig. 5a). To determine if the difference in pro-inflammatory gene expression was due to differences in aortic cellular composition, we analysed macrophage infiltration and activation in the artery wall of *Ldlr*^*−/−*^ mice transplanted with WT or *Angptl4*^*−/−*^ BM after 12 weeks on a WD. As shown in Fig. 5b, ANGPTL4 deficiency in haematopoietic cells markedly increased the accumulation of macrophages, which were identified as Lin-CD11b+F4/80^high^ cells, and inflammatory^8^ Ly-6C positive (Ly-6C^hi^) monocytes in the artery wall (Fig. 5b). These data demonstrate that ANGPTL4 deficiency in BM-derived cells results in increased vascular inflammation and elevated infiltration of monocyte/macrophages in the arterial wall of *Ldlr*^*−/−*^ mice.

Leukocytosis, in particular monocytosis and neutrophilia, is associated with increased cardiovascular risk in humans, and develops in hypercholesterolemic atherosclerotic animal models^46^,^47^. To investigate whether there was a difference in circulating leukocytes between *Ldlr*^*−/−*^ mice transplanted with BM from WT and *Angptl4*^*−/−*^ mice, we profiled the myeloid population in the peripheral blood before and after feeding a WD. Interestingly, we found a marked increase in the proportion of inflammatory Ly-6C^hi^ monocytes and neutrophils in the *Angptl4*^*−/−*^ BM transplanted group (Fig. 5c).

**Figure 6:**
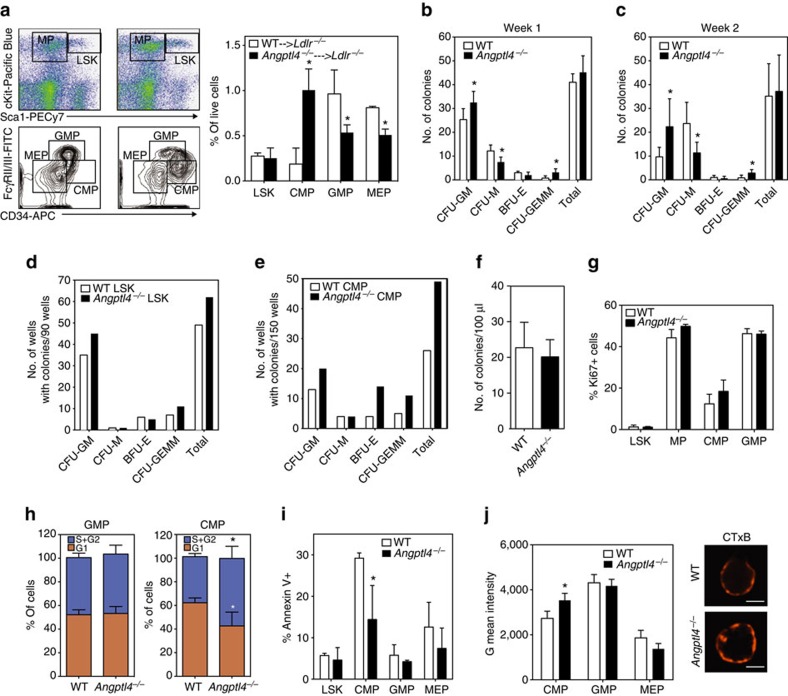
Haematopoietic ANGPTL4 deficiency promotes CMP expansion. (**a**) Dot plots showing the gating scheme of MP and LSK cells (upper panel), contour plots showing the gating schemes of GMP, CMP and MEP cells (lower panel) in the BM of *Ldlr*^*−/−*^ chimeras with WT or *Angptl4*^−/−^ BM on WD for 12 weeks. Right panel shows the quantification these progenitor populations (*n*=5 per group). Data are mean±s.e.m. *P<0.05 by comparison with data from *Ldlr*^*−/−*^ chimeras with WT BM by unpaired *t*-test. (**b**) Number of colonies from the BM isolated from WT and *Angptl4*^−/−^ mice using CFU assay. Cells were plated in methylcellulose media for 1 week; colonies were counted (**b**), resuspended and plated for 1 more week (**c**) (*n*=6 per group). Number of colony positive wells from LSKs (**d**) and CMPs (**e**) sorted from the BM and plated for 10 days individually in 96 well plates cells in methylcellulose media (*n*=4 per group; a pool of BM was used for sorting from each group). (**f**) Number of colonies from 100 μl blood from WT and *Angptl4*^−/−^ mice plated in methylcellulose media for 10 days (*n*=3 per group). (**g**) Proportion of Ki67-positive proliferating cells from indicated cell type from WT and *Angptl4*^−/−^ BM. (**h**) Cell cycle analysis of GMPs and CMPs from WT and *Angptl4*^−/−^ BM showing fraction of cells in G1 and S/G2 phase as determined by DAPI staining (*n*=5 per group). Cell cycle phase gates were drawn as approximations of the Watson (pragmatic) cell cycle modelling algorithm. (**i**) Proportion of annexin V positive apoptotic cells from indicated cell types from WT and *Angptl4*^−/−^ BM (*n*=5 per group). (**j**) Representative fluorescent images and flow cytometry quantification of lipid raft content in CMPs of WT and *Angptl4*^−/−^ BM assessed by CTxB staining (*n*=3 per group). Scale bars, 5 μm. All data are the mean±s.e.m. *P<0.05 by comparison with data from WT BM (**b**,**c**,**h**,**i**,**j**) by unpaired *t*-test.

To determine whether the increased circulating monocytes and neutrophils reflected a difference in the HSPC proliferation, we analysed BM populations in the *Ldlr*^*−/−*^ mice transplanted with BM from WT and *Angptl4*^*−/−*^ mice following 12 weeks of WD feeding. While there was no difference in the frequency of primitive Lin-Sca+c-Kit+ (LSK) cells, there was a markedly different distribution of the immediate precursors of leukocytes, myeloid progenitors (MP), between the groups (Fig. 6a). Within the MPs, *Angptl4*^*−/−*^ BM transplanted mice had a significantly higher frequency of CMPs and a lower frequency of granulocyte/monocyte progenitors (GMPs) and megakaryocyte/erythroid progenitors (MEPs) compared with WT BM transplanted mice (Fig. 6a). CMPs are the immediate precursors to GMPS, and sources of monocytes and neutrophils^48^. CMPs of *Angptl4*^*−/−*^ BM transplanted mice were highly positive for CD34, a cell surface marker for primitive haematopoietic stem cells (Fig. 6a). Taken together, these results demonstrate that ANGPTL4 deficiency in haematopoietic cells results in abnormal hematopoiesis and causes increase in CMP and leukocyte counts in *Ldlr*^*−/−*^ mice when fed a WD and contributes to the progression of atherosclerosis.

### ANGPTL4 deficiency promotes CMP expansion and survival

To gain insight into the mechanism of increased progenitor populations in *Angptl4*^*−/−*^ BM transplanted mice, first we checked the HSPC distribution in the BM of WT and *Angptl4*^*−/−*^ mice. *Angptl4*^*−/−*^ BM had higher frequency of CMPs and lower frequency of GMPs and MEPs without any significant difference in the frequency of LSK populations, suggesting that *Angptl4*^*−/−*^ animals have inherently aberrant progenitor population distribution. This was paralleled by an increase in the number of granulocyte monocyte colony-forming units (CFU-GM) and multipotent granulocyte, erythrocyte, monocyte, megakaryocyte CFUs (CFU-GEMM) that are capable of generating myeloid cells as assessed by colony-forming assays (Fig. 6b,c). Furthermore, in order to compare the proliferation potential of individual precursor cell types, CMPs and LSK cells from the WT and *Angptl4*^*−/−*^ BM were sorted, and single cells were used for colony-forming assays. Notably, higher numbers (62 out of 90) of *Angptl4*^*−/−*^ LSKs gave rise to colonies compared with WT LSKs (49 out of 90). The increase was more evident in CFU-GM and CFU-GEMM colonies (Fig. 6d). More importantly, almost twice as many *Angptl4*^*−/−*^ CMPs (49 out of 150) were capable of giving rise to colonies compared with WT CMPs (26 out of 150) with the difference more pronounced in CFU-GM, CFU-GEMM and BFU-E (erythrocyte, burst forming unit) (Fig. 6d). These results show the ability of *Angptl4*^*−/−*^ progenitors to grow into more primitive colonies, and suggest that they are more ‘stem-like' compared with WT progenitors. In contrast to the BM, there was no difference in the mobilization of the progenitor cells, as there was no difference in the number of colonies from the peripheral blood of WT and *Angptl4*^*−/−*^ mice (Fig. 6f).

To determine if the increased number of colonies in *Angptl4*^*−/−*^ was a result of increased proliferation, we assessed proliferation using Ki-67 staining. Although the number of actively proliferating Ki-67+ CMPs tended to be higher, it failed to reach significance (Fig. 6g). To further evaluate the proliferating cells, we performed cell cycle analysis of the progenitor cells. Interestingly, significantly more CMPs but not the GMPs within *Angptl4*^*−/−*^ BM were in S/G2 phase confirming the earlier observations that a higher number of *Angptl4*^*−/−*^ CMPs were undergoing proliferation (Fig. 6h). Since almost twice the number of *Angptl4*^*−/−*^ CMPs gave rise to colonies despite having only around 15% more cells in proliferative cell cycle, we hypothesized that *Angptl4*^*−/−*^ CMPs have some additional features that supported their survival. To test this hypothesis, we assessed apoptosis among progenitor cells using Annexin V staining. Interestingly, *Angptl4*^*−/−*^ CMPs were significantly less apoptotic than WT CMPs (Fig. 6i). These observations suggest that the leukocytosis observed in the *Angptl4*^*−/−*^ BM transplanted mice is possibly an exaggeration of existing differences in the HSPC population in *Angptl4*^*−/−*^ mice, whereby these mice have increased CMPs with higher proliferation potential and better survival and thus have the potential to give rise to more progenies including neutrophils and monocytes.

One of the possible mechanisms that can regulate HSPC proliferation is through signalling mediated from cell surface lipid rafts^49^. For example, excessive cholesterol accumulation in ABC transporter-deficient stem cells increases lipid rafts in stem cell membranes, resulting in increased surface localization of the IL-3/GM-CSF receptor and subsequent downstream signalling and cell proliferation in response to IL-3/GM-CSF^6^. We assessed whether there was a difference in lipid raft content in the stem cell progenitors of WT and *Angptl4*^*−/−*^ mice. Remarkably, there were more lipid rafts in the *Angptl4*^*−/−*^ CMPs as assessed by cholera toxin B (CTxB) staining, suggesting that the difference in proliferation and survival of CMPs is associated with increased lipid raft content (Fig. 6j).

### Macrophage ANGPTL4 deficiency increases foam cell formation

**Figure 7:**
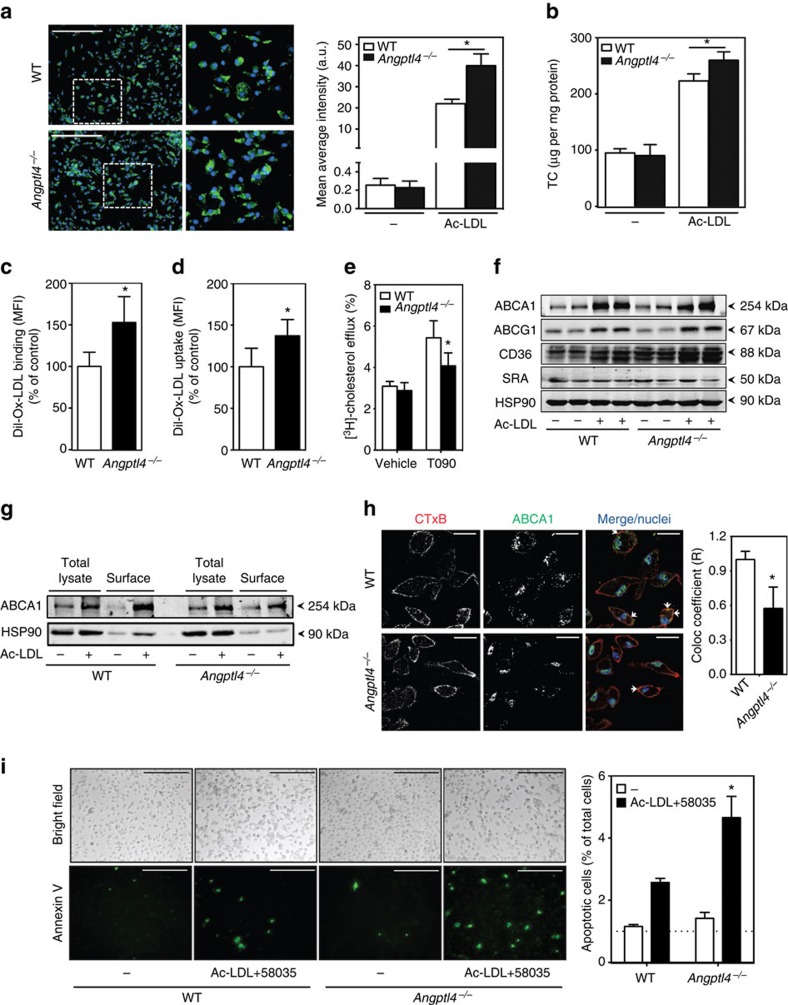
ANGPTL4 deficiency promotes macrophage foam cell formation and apoptosis. (**a**) Representative pictures from WT and *Angptl4*^*−/−*^ mouse peritoneal macrophages incubated with or without Ac- LDL (120 μg ml^−1^) for 24 h and stained with BODIPY 493/503 (1 μg ml^−1^) and DAPI (Green and blue, respectively). Scale bar, 5 μm. Quantification of the mean average intensity is in the right panel. (**b**) Total cholesterol content in peritoneal macrophages isolated from WT and *Angptl4*^*−/−*^ mice incubated with or without Ac-LDL (120 μg ml^−1^) for 24 h. (**c**) Flow cytometry analysis of DiI-Ox-LDL binding in peritoneal macrophages incubated with DiI-Ox-LDL (30 μg cholesterol per ml) for 30 min at 4 °C. At the end of the incubation period, cells were washed and incubated in RPMI 10% FBS media for 15 min at 37 °C to allow the internalization. (**d**) Flow cytometry analysis of DiI-Ox-LDL uptake in peritoneal macrophages incubated with DiI-Ox-LDL (30 μg cholesterol per ml) for 2 h at 37 °C. The results are expressed in terms of specific MFI after subtracting auto-fluorescence of cells incubated in the absence of DiI-Ox-LDL. (**e**) Cholesterol efflux to apolipoprotein A1 (ApoA1) in peritoneal macrophages isolated from WT and *Angptl4*^*−/−*^ mice stimulated with or without T0901317 (T090). (**f**) Western blot analysis of indicated proteins in peritoneal macrophages from WT and *Angptl4*^*−/−*^ mice incubated with or without Ac-LDL (120 μg ml^−1^) for 24 h. (**g**) Western blot analysis (representative of three blots) of ABCA1 expression in WT and *Angptl4*^*−/−*^ peritoneal macrophages incubated with Ac-LDL for 24 h. Surface ABCA1 was isolated using biotinylation followed by incubation with neutravidin. HSP90 is used as loading control (f and g). Full scans of westerns blots are provided in Supplementary Fig. 8. (**h**) Representative confocal images of mouse peritoneal macrophages from WT and *Angptl4*^*−/−*^ mice incubated with Ac-LDL for 24 h and stained with cholera toxin B (CTxB), ABCA1 and DAPI. Quantification of co-localization of CTxB and ABCA1 is on the right panel. Scale bar, 10 μm. (**i**) Representative images of WT and *Angptl4*^*−/−*^ macrophages cultured on coverslips and treated with or without Ac-LDL (120 μg ml^−1^) in combination with ACAT inhibitor (58035) for 24 h to induce lipid-loading-induced apoptosis (scale bars, 200 μm). Apoptosis was detected using Annexin-V staining. Right panel shows the quantification of percentage of apoptotic cells from four random fields from each cover slip. All data represent the mean±s.e.m. from at least three experiments in duplicate; *P<0.05 compared with WT macrophages by unpaired *t*-test. MFI, median intensity of fluorescence.

In order to explore additional mechanisms that could contribute to the accelerated atherosclerosis observed in *Angptl4*^*−/−*^ BM transplanted mice, we investigated the role of ANGPTL4 in the regulation of macrophage functions. Since ANGPTL4 expression is increased in macrophage-derived foam cells, we sought to determine whether ANGPTL4 influences lipid accumulation in macrophages. Interestingly, *Angptl4*^*−/−*^ macrophages accumulated significantly more neutral lipids and cholesterol compared with WT macrophages as assessed by bodipy staining and cholesterol oxidase assay respectively (Fig. 7a,b). We next investigated the mechanisms that could contribute to increased lipid accumulation in ANGPTL4-deficient macrophages. To this end, we evaluated DiI-Ox-LDL binding and uptake by flow cytometry. Absence of ANGPTL4 enhanced Ox-LDL binding and uptake (Fig. 7c,d). Moreover, there was decreased cholesterol efflux to ApoA1 in ANGPTL4-deficient macrophages stimulated with the LXR agonist T0901317 (Fig. 7e). These results suggest that both increased Ox-LDL uptake and decreased cholesterol efflux contributed to the enhanced lipid loading observed in *Angptl4*^*−/−*^ macrophages. Mechanistically, we found that CD36 but not SR-A1 expression levels were higher in *Angptl4*^*−/−*^ macrophages compared with WT macrophages (Fig. 7f). Surprisingly, despite the reduced cholesterol efflux in *Angptl4*^*−/−*^ macrophages, there was no difference in the total protein expression of ATP-binding cassette transporters ABCA1 and ABCG1, the main transporters responsible for cholesterol efflux in macrophages (Fig. 7f). This led us to investigate whether there was a difference in the distribution of ABCA1 between WT and *Angptl4*^*−/−*^ macrophages as the localization of ABCA1 in the cell surface is important in order to lipidate ApoA1 (ref. 50). Therefore, we checked the surface expression of ABCA1 using a biotinylation assay and immunofluorescence studies and found that there is less surface ABCA1 in *Angptl4*^*−/−*^ macrophages compared with WT macrophages (Fig. 7g,h). Taken together, these results demonstrate that loss of ANGPTL4 in macrophages enhances foam cell formation by regulating influx and efflux of lipoproteins and cholesterol in a reciprocal manner.

Foam cell death gives rise to large regions within plaques containing lipids and necrotic debris^51^,^52^. It is thought that free cholesterol accumulation in the endoplasmic reticulum (ER) results in ER stress-induce apoptosis^52^. Given our observation that ANGPTL4 deficiency promotes lipid accumulation in macrophages, we hypothesized that this could enhance macrophage apoptosis. To this end, we incubated *Angptl4*^*−/−*^ and WT macrophages with Ac-LDL and an acetyltransferase (ACAT) inhibitor (58035), to promote free cholesterol accumulation. Consistent with our hypothesis, cholesterol loading increases apoptosis in *Angptl4*^*−/−*^ macrophages assessed by Annexin V staining (Fig. 7i).

A continuum of pro- and anti-inflammatory macrophages, with extreme polarization phenotypes, M1 and M2, can be found in atherosclerotic lesions^53^. While M1-skewed macrophages promote inflammation and have been shown to be more abundant in the rupture-prone sections of atherosclerotic plaques, M2 macrophages are located in more stable, cell-rich areas of plaques and are associated with plaque resolution^54^,^55^. To investigate whether ANGPTL4 influences macrophage polarization, we incubated mouse BM-derived macrophages (BMDMs) with M1 or M2 polarizing cytokines, LPS/IFNγ or IL-4, respectively. The results showed that M1 polarized *Angptl4*^*−/−*^ BMDMs expressed higher mRNA levels of M1 markers including, *Nos2*, *Cox2* and *Tnfα* and protein levels of COX2 compared with WT BMDMs (Supplementary Fig. 5a,b). In contrast, the expression of *Ym1*, a well-established M2 marker, was less in *Angptl4*^*−/−*^ BMDMs after M2 polarization, while the expression of other M2 markers was not changed (Supplementary Fig. 5a).

Monocyte/macrophage migration is an important step in the early atherogenic process. Monocytes infiltrate atherosclerotic plaques in response to cytokines, such as macrophage chemoattractant protein-1 (MCP-1, also known as CCL2)^56^. In order to investigate the role of ANGPTL4 in this process, we assessed macrophage migration *in vitro*. As seen in Supplementary Fig. 6a,b, MCP-1-induced migration was unaltered in macrophages lacking ANGPTL4. In agreement with this, CCR2 expression was identical in blood monocytes from *Ldlr*^*−/−*^ mice transplanted with *Angptl4*^*−/−*^ and WT BM (Supplementary Fig. 6c). Collectively, these results demonstrate that in addition to increases in leukocyte count through CMP proliferation, ANGPTL4 deficiency also enhances macrophage foam cell formation, polarizes macrophages towards a M1 inflammatory phenotype, increases apoptosis susceptibility in response to cholesterol loading and promotes atherosclerosis.

## Discussion

**Figure 8:**
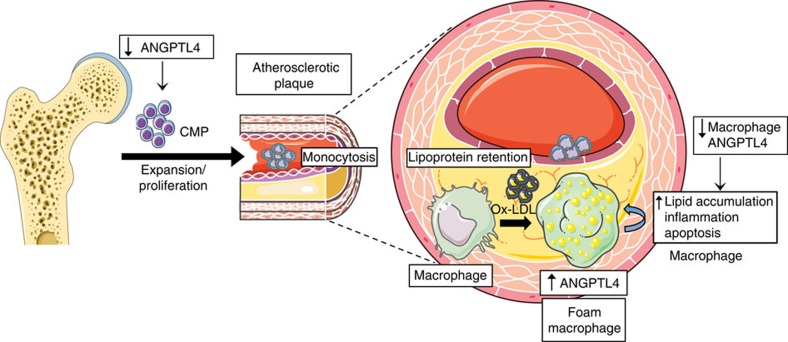
ANGPTL4 deficiency results in monocytosis and massive atherosclerosis. Schematic diagram showing haematopoietic ANGPTL4 function in atherosclerosis. ANGPTL4 is induced in macrophages in response to lipoprotein loading. ANGPTL4 deficiency in macrophages results in increase in foam cell formation, inflammation and apoptosis of macrophages within atherosclerotic plaques. Concomitantly, ANGPTL4 deficiency in haematopoietic cells results in an increase in the frequency and survival of CMPs, and upon WD feeding results in an elevated level of circulating monocytes. Overall, ANGPTL4 deficiency in haematopoietic cells promotes massive atherosclerosis.

In this study, we have identified an important protective role for macrophage-derived ANGPTL4 in the pathogenesis of atherosclerosis. While global depletion of ANGPTL4 results in several confounding metabolic abnormalities and inflammation, along with attenuated atherosclerosis, haematopoietic cell-specific ablation of ANGPTL4 using BM transplantation from *Angptl4*^*−/−*^ mice to *Ldlr*^*−/−*^ recipients results in a dramatic increase in atherosclerosis. Importantly, *Angptl4*^*−/−*^ BM transplanted mice have elevated blood leukocyte counts, higher frequency of Ly6-C^hi^ inflammatory monocytes and macrophages in aortic lesions, and increased vascular inflammation compared with mice transplanted with WT BM. Differences in leukocyte count seem to be associated with skewed progenitor population distribution, as the frequency of CMP population is higher in *Angptl4*^*−/−*^ animals. The CMPs from *Angptl4*^*−/−*^ mice have higher proliferative potential, increased lipid raft content and decreased apoptosis. This study provides a definitive role of haematopoietic ANGPTL4 during the progression of atherosclerosis by controlling leukocyte populations, and preventing lipid overloading and generation of macrophage foam cells (Fig. 8). This report demonstrates for the first time that ANGPTL4 can modulate selective HSPC proliferation, although several members of ANGPTL family with similar structures have been shown to stimulate HSPC expansion^9^,^11^,^57^,^58^.

We demonstrate that ANGPTL4 deficiency in haematopoietic cells accelerates atherosclerosis progression. Increased atherogenesis in haematopoietic ANGPTL4-deficient mice seems to have resulted from two distinct mechanisms- a higher leukocyte count and enhanced lipid loading/foam cell formation and subsequent apoptosis and inflammation. In response to hypercholesterolemia, the BM and spleen overproduce pro-inflammatory Ly-6C^hi^ monocytes that can infiltrate and preferentially accumulate and differentiate to macrophages in lesions^8^,^59^. Consistent with this, we found a higher frequency of leukocytes in *Ldlr*^*−/−*^ mice transplanted with *Angptl4*^*−/−*^ BM as a consequence of higher proliferation of CMPs in the BM. However, the frequency of GMPs, the immediate precursors of leukocytes, were lower in the *Angptl4*^*−/−*^ BM transplanted mice. The discrepancy in the ratios of GMPs, the immediate precursors of monocytes, and CMPs, the precursors of GMPs, in ANGPTL4-deficient mice is intriguing and needs further investigation. First, as the most direct precursors of monocytes, the GMPs might be differentiating to their progeny more quickly than usual. In other words, it is possible that the low GMP number reflects a faster transition from GMP to monocytes in the animals deficient in ANGPTL4. It is similarly possible that CMPs, whose production is heightened, do not replenish GMPs quickly enough. Thus, a combination of these processes would lead to the observed phenotype. Another possibility might be that CMPs do not go through the GMP intermediate at all. The hierarchical haematopoietic tree, where one intermediate must give rise to another before finally becoming a terminally differentiated leukocyte has been challenged by several recent studies showing that upstream progenitors can bypass downstream progenitors as they differentiate to leukocytes^60^. Although we have no evidence that this is the case, it is possible that CMPs differentiate to monocytes directly. Further studies are warranted to elucidate the mechanisms for the selective increase in CMP population observed in ANGPTL4-deficient BM.

Although previous studies have reported that ANGPTL4 prevents lipid overload in macrophages^27^, a comprehensive understanding of the mechanisms behind this process and subsequent impact on atherosclerosis has been lacking. We provide experimental evidence showing that ANGPTL4 promotes foam cell formation by concomitantly increasing modified LDL uptake and decreasing cholesterol efflux. The increase in uptake seems to be mediated through at least two mechanisms- increased CD36 expression and inhibition of LPL activity. We found that CD36 expression is higher in *Angptl4*^*−/−*^ macrophages and is further enhanced with Ac-LDL. CD36 expression is regulated by modified LDL particles and free fatty acids^61^. As such, enhanced lipid loading in *Angptl4*^*−/−*^ macrophages seems to follow a positive feedback response, whereby increased CD36 on the cell surface leads to more lipoprotein loading and fatty acid release and this in turn leads to increased CD36 expression. LPL is also known to facilitate retention and uptake of lipoproteins by cell surface receptors through its bridging activity^62^. In fact, macrophage-specific overexpression of LPL results in the accumulation of macrophage foam cells and atherosclerotic lesion formation, whereas LPL deletion results in the opposite^28^,^63^. Since ANGPTL4 is a strong inhibitor of LPL, enhanced modified LDL uptake in ANGPTL4-deficient macrophages might also be mediated through increased activity of LPL. However, the fact that transgenic mice expressing catalytically active or inactive LPL in macrophages show similar atherogenic effects suggests that the bridging function of LPL is sufficient to drive its effect in macrophages^64^. In a recent study, ANGPTL4 treatment resulted in more pronounced reduction in Ox-LDL uptake by macrophages compared with Orlistat, an inhibitor of LPL activity, indicating that ANGPTL4 possibly also regulates non-catalytic bridging activity of LPL^31^. Further investigation using LPL and ANGPTL4-deficient macrophages is required to confirm this possibility.

In addition to a significant reduction in Ox-LDL uptake, we also demonstrate a marked decrease in cholesterol efflux in *Angptl4*^*−/−*^ macrophages. Importantly, we found that absence of ANGPTL4 influences ABCA1 expression in the cell surface. Although it is not clear how the absence of ANGPTL4 influences the cellular localization of ABCA1, a previous report has shown that elevated unsaturated fatty acids such as palmitoleate and oleate, which are enriched in macrophage foam cells^65^, decrease ABCA1 in plasma membrane^66^. Thus, it is possible that absence of ANGPTL4 could increase intracellular unsaturated fatty acid content leading to a reduction in ABCA1 cell surface expression.

In summary, this study demonstrates that ANGPTL4 can have profound effects on quantitative and qualitative properties of macrophages. On the one hand, ANGPTL4 appears to regulate the net blood monocyte content in lipid-rich conditions by restricting the expansion of precursors of monocytes, CMPs. On the other hand, ANGPTL4 suppresses lipid overloading in macrophages and prevents generation of foam cells and inflammation. Altogether, by controlling these critical myeloid features, haematopoietic ANGPTL4 reduces the progression of atherosclerosis. The findings from this study suggest that interventions capable of enhancing ANGPTL4 expression, specifically in macrophages, may lessen plaque progression.

## Methods

### Animals

Male C57BL/6 (WT) and *Ldlr*^*−/−*^ mice were purchased from Jackson Laboratories (Bar Harbor, ME) and kept under constant temperature and humidity in a 12 h controlled dark/light cycle. *Angptl4*^*−/−*^ mice were provided by the laboratory of Andras Nagy (Samuel Lunenfeld Research Institute, Mount Sinai Hospital). *Angptl4*^*−/−*^ mice that have been backcrossed eight generations onto a C57BL/6 background were crossed with *Ldlr*^*−/−*^ mice, also on the C57BL/6 background, so that mice heterozygous at both loci could be generated. These *Angptl4*^*+/−*^ *Ldlr*^*+/−*^ mice were crossed a second time with *Ldlr*^*−/−*^ mice. The *Angptl4*^*+/−*^*Ldlr*^*−/−*^ progeny from this round of breeding were then intercrossed, producing *Angptl4*^*−/−*^*Ldlr*^*−/−*^ and *Angptl4*^*+/+*^*Ldlr*^*−/−*^ littermates that were used as controls for all studies. Male mice were used for experiments after they were 8–10 weeks old. Accelerated atherosclerosis was induced by feeding the mice for 12 weeks with a WD containing 1.25% cholesterol (ResearchDiets, D12108). All the experiments were approved by the Institutional Animal Care Use Committee of New York University and Yale University School of Medicine.

### mRNA microarray analysis

Mouse thioglycollate-elicited peritoneal macrophages we incubated in presence of Ac-LDL (120 μg ml^−1^) for 24 h. Total RNA was extracted using TRIzol (Invitrogen), and mRNA was purified using and mRNA isolation Kit (Qiagen). The purity and integrity of total RNA sample was verified using the Agilent Bioanalyzer (Agilent Technologies, Santa Clara, CA). mRNA was hybridized to Illumina expression profiling microarray (Mouse WG-6 v2.0 Expression beads Chip) according to the manufacturer's directions. The data discussed in this publication have been deposited in NCBÍs Gene Expression Omnibus and are accessible through GEO Series accession number GSE83090 (https://www.ncbi.nlm.nih.gov/geo/query/acc.cgi?acc=GSE3090). Raw data were normalized and analysed by GeneSpring GX software version 11.5 (Agilent Technologies).

### Lipids and lipoprotein profile measurements

Mice were fasted for 12–14 h before blood samples were collected by retro-orbital venous plexus puncture. Then, plasma was separated by centrifugation and stored at −80 °C. Total plasma cholesterol and TGs were enzymatically measured (Wako Chemicals, USA) according to the manufacture's instructions. The lipid distributions in plasma lipoprotein fractions were assessed by fast-performed liquid chromatography gel filtration with 2 superose 6 HR 10/30 columns (Pharmacia).

### Histology and morphometric analyses

Mouse hearts were perfused with 10 ml of PBS and were put in 10 ml of 4% paraformaldehyde overnight. After incubation in PFA, hearts were washed with PBS and left with PBS for 1 h. Next, hearts were put in 30% sucrose until the next day. Finally, hearts were embedded in OCT and frozen. Serial sections were cut at 8 μm thickness using a cryostat. Every third slide from the serial sections was stained with haematoxylin and eosin (H&E) and each consecutive slide was stained with oil red O (ORO) for quantification of lesion area. Aortic lesion size of each animal was obtained by averaging the lesion areas in four sections from the same mouse. The necrotic core area was measured as a percentage of the total plaque area from the three sections from the same mouse. Collagen content was assessed by Picrosirius red-staining of consecutive slides from serial sections. CD68 staining (1:200; Serotec; #MCA1957) was used as a macrophage marker using consecutive slides from serial sections. Apoptotic cells in lesions were detected by TUNEL after proteinase K treatment, using *in situ* Cell Death Detection kit, TMR red (Roche) according to the manufacturer's instructions. Nuclei were counterstained with DAPI for 10 min. The data are expressed as the number of TUNEL-positive cells per mm2 cellular lesion area. Proliferating cells in each lesion were detected by Ki-67 staining (1:100; Abcam; #66155). Percentage of proliferating cells was calculated as the number positive Ki-67-labelled nuclei divided by the number of DAPI-stained nuclei. In separate sections from *Ldlr*^*−/−*^ mice on high-cholesterol diet, ANGPTL4 (1:100; Invitrogen; #40-9800) and CD68 expression were detected using immunofluoresecence.

### Human atherosclerotic plaques

Ten atherosclerotic plaques (stages V–VI) from patients undergoing carotid endarterectomy in our institution were fixed with paraformaldehyde and embedded in paraffin for histological analysis. In addition, 10 carotid endarterectomy samples were dissected separating the stenosis CP from the non-complicated (NCP) fibrous adjacent area as previously described^62^. The CP was defined as the lesion, usually localized at the origin of the internal carotid artery responsible for the surgery. The CP contained an important proportion of inflammatory cells (Stary stages V–VI), whereas the NCP adjacent areas were mainly composed of VSMCs (Stary stage III). These tissues were snap frozen in liquid nitrogen and homogenates were divided and resuspended for mRNA and protein analysis. The study was approved by the hospital's ethics committee (IIS-Fundación Jiménez Díaz) according to the institutional and the Good Clinical Practice guidelines and was performed in accordance with the Declaration of Helsinki. Written informed consent was obtained from patients undergoing carotid endarterectomy and samples were kept under the IIS-FJD biobank supervision [Ministerio de Sanidad y Consumo, Instituto de Salud Carlos III, biobancos (RD09/0076/00101)].

### Immunohistochemistry of carotid endarterectomy plaques

The carotid atherosclerotic plaques were fixed with PFA and embedded in paraffin for histological analysis and were then cross-sectioned into 4 μm thick pieces, dewaxed, and rehydrated. Immunohistochemical staining was assessed using anti-CD68 (1:250; Dako; clone kp1, M0814), or anti- ANGPTL4 (1:100; Thermo Scientific; #PA5-26216) antibodies, followed by mouse secondary antibody. ABComplex/HRP (Vector Laboratories) was added, and sections were stained with 3,3′-diaminobenzidine (Dako). Corresponding hematoxylin staining was used for nucleus identification. Negative controls using the corresponding IgG were included for checking nonspecific staining.

### Bone marrow transplantation

Eight-week-old male *Ldlr*^*−/−*^ mice were lethally irradiated with double dose of 550 rads (5.5 Gy) from a caesium source 4 h apart before transplantation. BM was collected from femurs of donor *Angptl4*^*−/−*^ or *WT* mice by flushing with sterile medium (RPMI 1640, 2% fetal bovine serum (FBS), 5 U ml^−1^ heparin, 50 U ml^−1^ penicillin and 50 μg ml^−1^ streptomycin). Each recipient mouse was injected with 2 × 10^6^ BM cells through retro-orbital injection. Four weeks after BM transplantation, peripheral blood was collected by retro-orbital venous plexus puncture for PCR analysis of BM reconstitution. For atherosclerosis study, mice were fed with WD for 12 weeks beginning 4 weeks after BM transplantation. At the end of 12 weeks, mice were killed and lipid analysis and atherosclerotic lesion analysis were performed.

### Cell culture and treatments

Mouse macrophages (RAW 264.7) cells were obtained from American Type Tissue Collection (ATTC TIB-71). RAW 264.7 were grown in DMEM supplemented with10% FBS and 1% penicillin-streptomycin, and L-glutamine. Peritoneal macrophages from adult male C57BL/6J mice were harvested by peritoneal lavage four days after intraperitoneal injection of thioglycollate (3% w/v). Cells were plated in RPMI 1640 supplemented with 10% FBS, 100 units ml^−1^ penicillin and 100 units ml^−1^ streptomycin. After 4 h, non-adherent cells were washed out, and macrophages were incubated in fresh medium containing DMEM, 10% FBS, and 20% L-929 cells conditioned medium for 24 h and cells were maintained in culture as an adherent monolayer. Peritoneal macrophages were used for different experiments.

For BMDMs isolation, cells were flushed out from femur and tibia of WT and *Angptl4*^*−/−*^ mice, filtered with 40 μm cell strainer (BD Falcon) and resuspended at 6–8 × 10^6^ cells ml^−1^ in IMDM media (Invitrogen) supplemented with 10% FBS at room temperature (RT). The cell suspension was then layered over lympholyte M solution (Cedarlane) at the proportion of 1 ml per 10^6^ cells. After centrifugation at 1300*g* for 20 min at RT, mononuclear cells were collected from the interface. Cells were washed in Ca^2+^- and Mg^2+^-free PBS and resuspended in medium containing Iscove's DMEM, 20% FBS, 20% L-929 cells conditioned medium and 0.25 μg ml^−1^ fungizone and plated at a density of 1.7 × 10^5^ cells per ml on non-tissue coated plates. After 6 days in culture, non-adherent cells were eliminated and adherent cells were harvested for experiments.

### Cell migration assay

Cell migration assays were performed with a modified Boyden chamber with Costar Trans-well inserts (Corning). The inserts were coated with a solution of 0.1% gelatin. Sub-confluent BMDMs from WT and *Angptl4*^*−/−*^ mice were serum starved for 6 h. Solutions of MCP-1 (100 ng ml^−1^), M-CSF (100 ng ml^−1^), and 0.1% BSA were prepared in Dulbecco's modified Eagle's medium (DMEM) and added to the bottom chambers. Macrophages (7.5 × 10^4^ cells) were added to the upper chambers. After 16 h incubation at 37 °C, cells on both sides of the membrane were fixed and stained with the Diff-Quik staining kit (Baxter Health). Cells on the upper side of the membrane were removed with a cotton swab. The average number of cells per field on the lower side of the membrane from four high-power (× 400) fields was counted. Macrophage chemotaxis was also quantified using a Real-time Cell Invasion and Migration (RT-CIM) × CELLigence assay system with monitoring every 5 min (Roche Applied Science). Cell migration in response to MCP-1 (100 ng ml^−1^) was measured till 20 h.

### Cellular cholesterol measurement and foam cell formation assays

Thioglycollate-elicited peritoneal macrophages from WT and *Angptl4*^*−/−*^ mice were plated on 12-well plates and incubated with or without Ac-LDL (120 μg ml^−1^). After 24 h, intracellular cholesterol content was measured using the Amplex Red Cholesterol Assay Kit (Molecular Probes, Life Technologies), according to the manufacturer's instructions. Cholesterol ester was estimated by subtracting free cholesterol from total cholesterol. For the foam cell formation assay, peritoneal macrophages were grown on glass coverslips and treated with or without Ac-LDL (120 μg ml^−1^) for 24 h. Cells were then fixed with 4% PFA for 20 min. After washing three times with 1 × PBS, cells were blocked with 1% BSA in PBS for 30 min and stained with BODIPY 493/503 (Life Technologies) for 30 min at RT. Next, cells were washed twice with 1x PBS and stained the nuclei with DAPI for 10 min to visualize the nuclei. Finally, cells were mounted on glass slides with Prolong-Gold (Life Technologies). From each cover slip, four random fields were selected and images were acquired using Zeiss Axiovert 2000 M fluorescence microscope (Carl Zeiss). All gains for the acquisition of comparable images were maintained constant. Analysis of different images was performed using ImageJ (NIH) and Adobe Photoshop CS5.

### Cholesterol efflux assays

Peritoneal macrophages from WT and *Angptl4*^*−/−*^ mice were seeded at a density of 1 × 10^6^ cells per well 1 day before loading with 0.5 μCi per ml [^3^H]-cholesterol for 24 h with or without T0901317 (3 μmol l^−1^) for 12 h. Cells were then washed twice with PBS and incubated in RPMI 1640 medium supplemented with 2 mg ml^−1^ fatty acid free bovine serum albumin in the presence acetyl-coenzyme A ACAT inhibitor (2 μmol l^−1^; Novartis Corporation, New York, NY, USA) for 4 h before the addition of 50** **μg ml^−1^ human ApoA1 in FAFA media. Supernatants were collected after 6 h and expressed as a percentage of [^3^H]-cholesterol in the media per total cell [^3^H]-cholesterol content (total effluxed [^3^H]-cholesterol+cell-associated [^3^H]-cholesterol).

### Apoptosis assay

For cell death assay, mouse peritoneal macrophages were grown on coverslips and incubated with Ac- LDL (120 μg ml^−1^) and ACAT inhibitor (58035, 10 μg ml^−1^). After 24 h, cells were washed with Annexin V buffer and stained with FITC-conjugated Annexin V (1:20, Biolegend) and DAPI for 15 min in dark. From each cover slip, four random fields were selected and images were captured using Zeiss Axiovert 2000 M fluorescence microscope (Carl Zeiss). Quantification of the images was performed using Image J.

### Luciferase assay

PPRE in the intron 3 of *Angptl4* initially cloned into SEAP vector (gift of Sander Kersten, Wageningen University, The Netherlands) was excised out and cloned into pGL3 vector (Promega) downstream of firefly luciferase reporter gene. Raw macrophages were co- transfected with pGL3 construct (400 ng per well) and *Renilla* luciferase control plasmid (phRL-CMV; Promega, 1.5 ng per well) in 24-well plates using Lipofectamine 2000 (Invitrogen) according to the manufacturer's guide. 24 h after the transfection, cells were treated with or without Ac-LDL or rosiglitazone (a synthetic PPAR-γ agonist) for another 24 h. Luciferase levels were measured using Dual Luciferase Reporter Assay System (Promega). Firefly luciferase activity was normalized to the corresponding *Renilla* luciferase activity and plotted as a percentage of the control. Experiments were performed in triplicate and repeated at least three times.

### Blood leukocytes and aortic cells analysis

Blood was collected by retro-orbital puncture in heparinized micro-hematocrit capillary tubes. Erythrocytes were lysed with ACK lysis buffer (155 mM Ammonium Chloride, 10 mM Potassium Bicarbonate, 0.01 mM EDTA, pH 7.4). WBC were resuspended in 3% FBS in PBS, blocked with 2 μg ml^−1^ of FcgRII/III, then stained with a cocktail of antibodies. Monocytes were identified as CD115hi and subsets as Ly6-C^hi^ and Ly6-C^lo^; neutrophils were identified as CD115^lo^Ly6-C^hi^Ly6-G^hi^. The following antibodies were used (all from BioLegend): FITC-Ly6-C (AL-21), PE-CD115 (AFS98), APC- Ly6-G (1A8). All antibodies were used at 1:300 dilutions.

For the analysis of leukocytes in aortic tissue, entire aorta was perfused with PBS, cut in small pieces and subjected to enzymatic digestion with 400 U ml^−1^ collagenase I, 125 U ml^−1^ collagenase XI, 60 U ml^−1^ DNase I and 60 U ml^−1^ hyaluronidase (Sigma-Aldrich) for 1 h at 37 °C while shacking. Total viable cell numbers were obtained using Trypan Blue (Cellgro).

### Haematopoietic stem cells analysis

BM was harvested from femurs and tibias. Cells were blocked with 2 μg ml^−1^ of FITC-conjugated FcgRII/III and stained for lineage markers (B220 (RA3-6B2), CD11b (M1/70), Gr-1 (RB6-8C5), TER- 119, CD8a- (536.7), CD4 (RM4-5), IL7R (A7R34), all APC-Cy7-conjugated), BV421-ckit (2B8), PE-Cy7-Sca1 (D7), APC-CD34 (RAM34). CMPs were identified as Lin-/ckit+ Sca1-/CD34int, FcgRII/IIIint, while GMP as Lin-/ckit+ Sca1-/CD34int, FcgRII/IIIhi. All the antibodies were from BioLegend and antibody dilutions were 1:300-1:500. Flow cytometry was performed using a BD LSRII and flow cytometry and imaging were performed using Amnis Imagestream-X MarkII imaging flow cytometer. All flow cytometry data was analysed using FlowJo software (Tree Star) and imaging from flow cytometry was analysed using IDEAS application software. Apoptotic cells were analysed using Annexin V staining (1:20, Biolegend) and proliferative cells were identified using Ki-67 (1:300, Biolegend) and DAPI stainings.

### GM-CFU assay

BM was isolated from the femur of WT and *Angptl4*^*−/−*^ mice. Total of 2 × 10^4^ BM cells were plated in 1 ml methylcellulose-based media that was supplemented with an assortment of recombinant cytokines (Methocult GF M3434; StemCell Technologies) in 35 mm culture dishes. The number of GM-CFUs per dish was counted after 1 week, colonies were resuspended and 2 × 10^4^ cells from the suspension were replated in fresh methylcellulose-based media. Colonies were counted after another week in the media. In another set of experiments, CMP, GMP and LSK cells were sorted from the BM of WT and *Angptl4*^*−/−*^ mice using BD FACSAria cell sorter. Before sorting, HSPCs were enriched using mouse progenitor cell enrichment kit (Stem Cell Technologies). Identification scheme for different progenitor population for sorting was similar to the one used above to analyse progenitor cells. Single cells from LSK and CMPs were plated in 96 well plates in methylcellulose-based media. Total number of wells with colonies and types of colonies were evaluated after 10 days.

### Dil-Ox-LDL uptake and binding assays

DiI-Ox-LDL lipoproteins were oxidized and labelled with the fluorescent probe DiI (Molecular Probes, Invitrogen). For the uptake assays, mouse peritoneal macrophages were washed once in 1X PBS and incubated in fresh media containing DiI-Ox-LDL (30 μg cholesterol per ml) for 2 h at 37 °C. For the binding assays, cells were incubated for 15 min at 4 °C to stop membrane internalization. Then, cells were treated with fresh media containing Dil-Ox-LDL (30 μg cholesterol per ml) for 1 h and 30 min at 4 °C. At the end of the incubation period, cells were washed and 1 ml of RPMI 10% FBS added for 15 min at 37 °C to allow the internalization. Finally, in both assays, cells were washed and resuspended in 1 ml of PBS and analysed by flow cytometry (FACScalibur, Becton Dickinson). The results are expressed in terms of specific median intensity of fluorescence (M.I.F.) after subtracting auto-fluorescence of cells incubated in the absence of DiI-LDL.

### M1 and M2 polarization assay

BMDMs from WT and *Angptl4*^*−/−*^ mice were stimulated for 8 h with IL-4 (15 ng ml^−1^) (R&D) and LPS (10 ng ml^−1^) (Sigma) plus IFNγ (20 ng ml^−1^) (R&D) to polarize macrophages to M2 or M1, respectively. At the end of the treatment, cells were extensively washed with 1x PBS and RNA and protein were isolated.

### Western blotting

Tissue or cells were lysed in ice-cold buffer containing 50 mM Tris-HCl, pH 7.5, 125 mM NaCl, 1% NP- 40, 5.3 mM NaF, 1.5 mM NaP, 1 mM orthovanadate and 1 mg ml^−1^ of protease inhibitor cocktail (Roche) and 0.25 mg ml^−1^ AEBSF (Roche). Tissue or cell lysates were rotated at 4 °C for 1 h before the insoluble material was removed by centrifugation at 12000*g* for 10 min. After normalizing for equal protein concentration, cell lysates were resuspended in SDS sample buffer before separation by SDS–polyacrylamide gel electrophoresis. Following overnight transfer of the proteins onto nitrocellulose membranes, membranes were probed using the following antibodies (catalogue numbers and dilutions used are indicated within parentheses): rabbit polyclonal antibodies against ANGPTL4 (#40-9800; 1:100), ABCG1 (#NB400-132; 1:1000), COX-2 (#160106; 1:1000) and iNOS (#2982; 1:1000) were obtained from Invitrogen, Novus, Cayman and Cell Signaling Technology respectively; a mouse monoclonal antibody against ABCA1 (clone AB.H10; # ab18180, 1:1000) and a rabbit polyclonal antibody against CD36 (#ab124515; 1:1000) were obtained from Abcam; a goat polyclonal antibody against SR-A (#sc-20444; 1:1000) was from Santa Cruz and mouse monoclonal antibodies against HSP-90 (clone 68/HSP90; #610419, 1:1000) and GAPDH (clone GT239; #GTX627408, 1:5000) were purchased from BD Biosciences and GeneTex, respectively. Blots were then washed and incubated with fluorescently labelled secondary antibodies (Invitrogen). Protein bands were visualized using the Odyssey Infrared Imaging System (LI-COR Biotechnology). Densitometry analysis of the gels was carried out using ImageJ software from the NIH (http://rsbweb.nih.gov/ij/).

### Biotinylation of cell surface proteins

Biotinylation of surface protein was performed as explained before. Briefly, thioglycollate-elicited peritoneal macrophages from WT and *Angptl4*^*−/−*^ mice were plated on six-well plates and incubated with or without Ac-LDL (120 μg ml^−1^) for 24 h. Cells were then washed with PBS^++^ (1 × PBS supplemented with 0.02 mM CaCl_2_ and 0.15 mM MgCl_2_) and incubated for 30 min on ice with 250 μM EZ-link SulfoNHS-SS Biotin diluted in PBS++ (Life Technologies). Cells were again washed with PBS^++^ and the reaction was quenched for 30 min on ice in quenching buffer (PBS++ supplemented with 100 mM glycine). Cells were scraped and 1/5^th^ of suspension were set aside as whole lysate and rest of the biotin-modified proteins were immunoprecipitated with NeutrAvidin agarose beads (Life Technologies) overnight at 4 °C. Biotin-modified proteins were collected by centrifugation at 5,000*g* for 5 min. Intracellular, unmodified proteins were collected from the supernatant of the 5,000*g* spin. The streptavidin beads were washed three times in PBS^++^ before proteins were removed from the beads by incubation at 42 °C for 20 min, in 2 × SDS sample loading buffer supplemented with β-mercaptoethanol. Biotinylated cell surface ABCA1 was detected by immunoblotting total surface proteins against ABCA1 antibody (1:1000; Abcam; clone AB.H10; # ab18180).

### Fluorescence microscopy

For ABCA1 localization experiments, thioglycollate-elicited peritoneal macrophages were plated on coverslips and incubated with Ac-LDL (120 μg ml^−1^) for 24 h. At the end of incubation, cells were washed with PBS and incubated with fluorescently labelled cholera toxin B (CTxB) in 0.1% BSA in PBS at 4 °C for 20 min. Cells were then fixed with 4% PFA on ice for 10 min, permeabilized with 100% pre-chilled methanol at −20 °C for 2 min, blocked with 4% goat serum in PBS for 1 h and stained with monoclonal antibody against ABCA1 (1:1000; Abcam; clone AB.H10; # ab18180) overnight. After three washes with 1 × PBS, cells were incubated with Alexa Fluor 488 donkey anti-mouse secondary antibody (1:250, life technologies; # A21202) for 1 h at RT and coverslips were mounted on glass slides with mounting media with DAPI (Vectashield, Vector Laboratories; # H-1200). All images were analysed using a confocal microscope (Leica SP5 II) equipped with a 63X Plan Apo Lenses. All gains for the acquisition of comparable images were maintained at a constant level. Analysis of different images was performed using ImageJ (NIH) and Adobe Photoshop CS5.

### RNA isolation and quantitative real-time PCR

Total RNA from human plaques or mouse aortas was isolated using the Bullet Blender Homogenizer (Next Advance) in TRIzol reagent (Invitrogen) according to the manufacturer's protocol. Total RNA from BMDM was isolated using TRIzol reagent. 1 μg of total RNA was reverse transcribed using the iScript RT Supermix (Bio-Rad), following the manufacturer's protocol. Quantitative real-time PCR was performed in triplicate using SsoFast EvaGreen Supermix (BioRad) on a Real-Time Detection System (Eppendorf). The mRNA level was normalized to 18 s as a housekeeping gene (Primers, Supplementary Table 1).

### Statistical analysis

Animal sample size for each study was chosen on the basis of literature documentation of similar well-characterized experiments^67^,^68^. The number of animals used in each study is listed in the figure legends and in the main text. No inclusion or exclusion criteria were used and studies were not blinded to investigators or formally randomized. *In vitro* experiments were routinely repeated at least three times unless otherwise noted. All data are expressed as mean±s.e.m. Statistical differences were measured using an unpaired two-sided Student's *t-*test. Normality was checked using the Kolmogorov–Smirnov test. A nonparametric test (Mann–Whitney) was used when data did not pass the normality test. A value of *P*≤0.05 was considered statistically significant. Data analysis was performed using GraphPad Prism Software Version 5.0a (GraphPad, San Diego, CA).

### Data availability

All relevant data are available from the authors. The microarray data have been deposited in the NCBI Gene Expression Omnibus data base GSE83090.

## Additional information

**How to cite this article:** Aryal, B. *et al.* ANGPTL4 deficiency in hematopoietic cells promotes monocyte expansion and atherosclerosis progression. *Nat. Commun.* 7:12313 doi: 10.1038/ncomms12313 (2016).

## Supplementary Material

Supplementary Information Supplementary Figures 1-9 and Supplementary Table 1
