# Supplementary material for: ANGPTL4 deficiency in haematopoietic cells promotes monocyte expansion and atherosclerosis progression
Source: Nat Commun. 2016 Jul 27;7:12313. doi: 10.1038/ncomms12313 (PMC4974469; doi:10.1038/ncomms12313)
Supplement: Supplementary Information — Supplementary Figures 1-9 and Supplementary Table 1 [file ncomms12313-s1.pdf]

Supplementary Figure 1

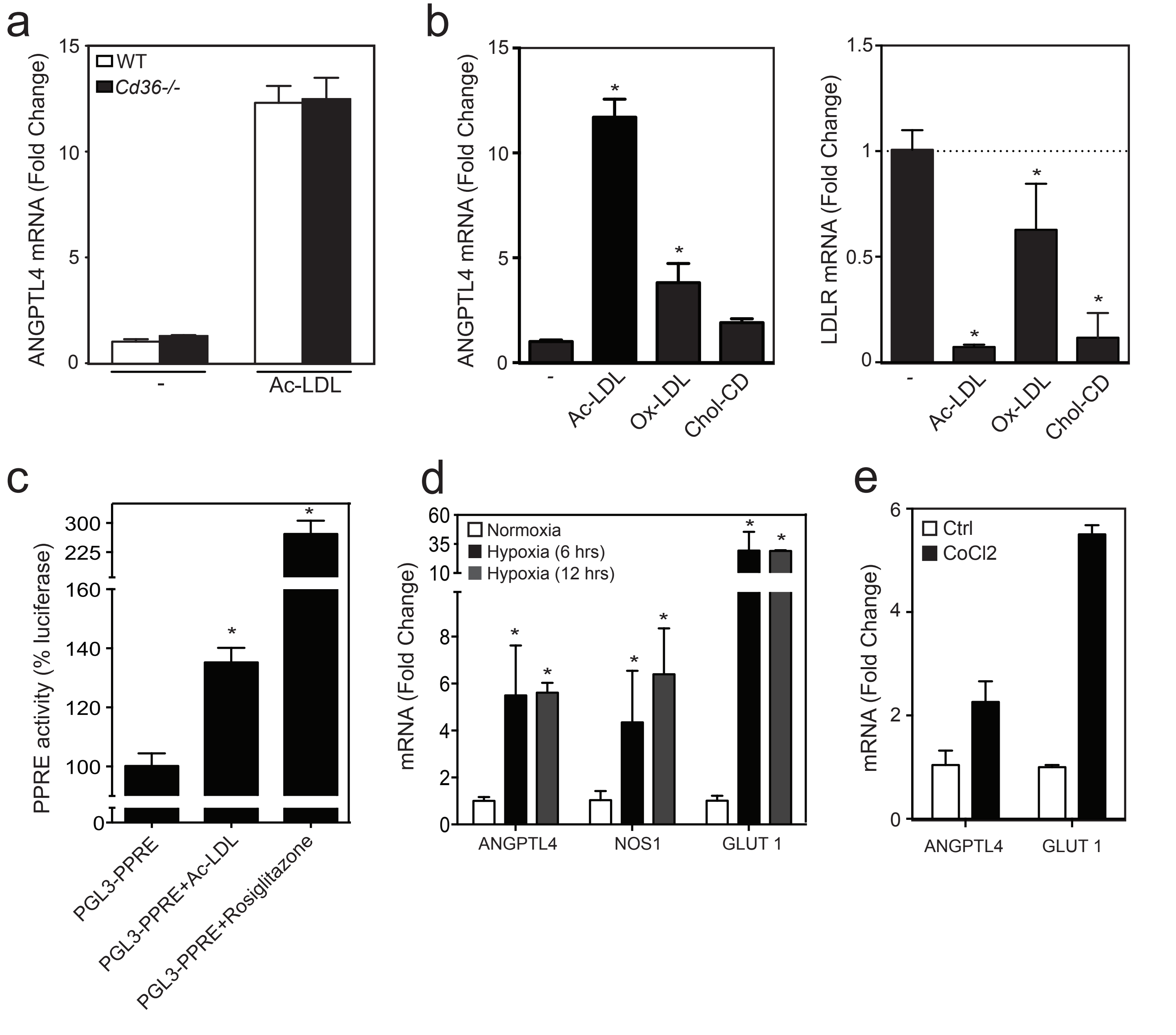

Supplementary Fig. 1. ANGPTL4 expression in macrophages is regulated by PPARs and hypoxia. (**a**) Angptl4mRNA expression in peritoneal macrophages from WT and Cd36$^{-/-}$ mice treated with or without Ac-LDL (120 µg/ml) for 24 h. (**b**) Angptl4 mRNA expression in mouse peritoneal macrophages with indicated treatments for 24 h. Right panel shows the expression of Ldlr as a control for cholesterol loading. (**c**) Luciferase activity assay in Raw 264.7 cells transfected with PPAR response element from mouse Angptl4 linked to firefly luciferasereporter construct along with renilla luciferase vector and incubated with or without indicated treatments for 24 h. Rosiglitazone is used as a positive control for induction of PPAR activity. (**d**) Angptl4 mRNA expression in mouse peritoneal macrophages incubated in hypoxic condition (1% O_2) for indicated time points. Glut1 and Nos2 mRNA expression are used as positive controls for hypoxia. (**e**) mRNA expression of Angptl4 in mouse peritoneal macrophages incubated with or without chemical inducer of hypoxia cobalt chloride ($CoCl_2$) for 12 h. Glut1 expression is used as a positive control for $CoCl_2$ treatment. All data represent the mean ± SEM and are representative of 3 experiments in duplicate. *P ≤ 0.05, Student's t-test.

Supplementary Figure 2

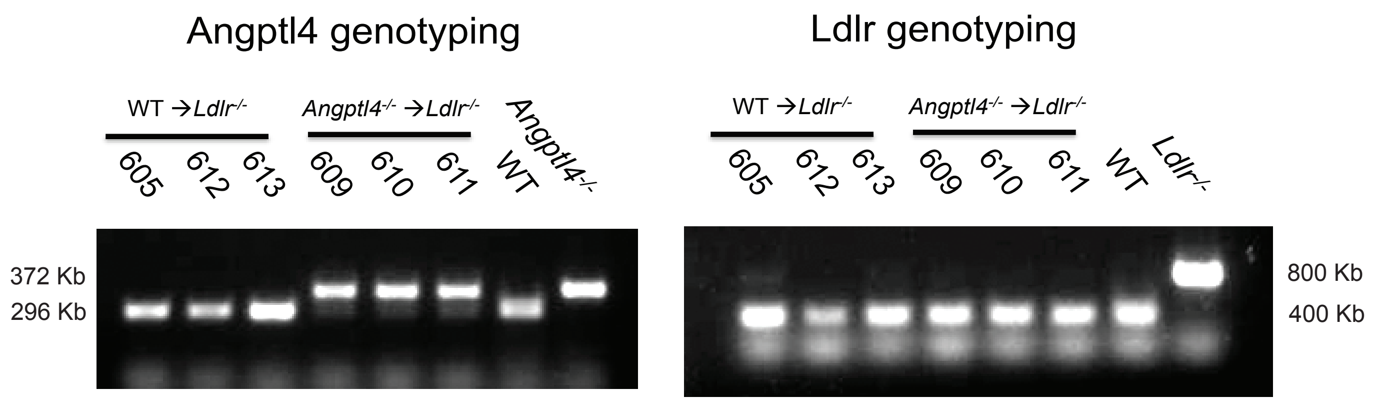

Supplementary Fig. 2. Genotyping of Ldlr−/− chimeras with WT or Angptl4−/− BM. PCR amplification of Angptl4 (left panel) and Ldlr (right panel) genes. Genomic DNA prepared from peripheral blood of Ldlr−/− chimeras with WT or Angptl4−/− BM were amplified using specific primers for Angptl4 or Ldlr. In each panel, lanes 1, 2, and 3 are from Ldlr−/− chimeras with WT BM, lanes 4, 5, and 6 are from Ldlr−/− chimeras with Angptl4−/− BM, lane 7 is from WT and lane 8 from Angptl4−/− (left) or Ldlr−/− (right).

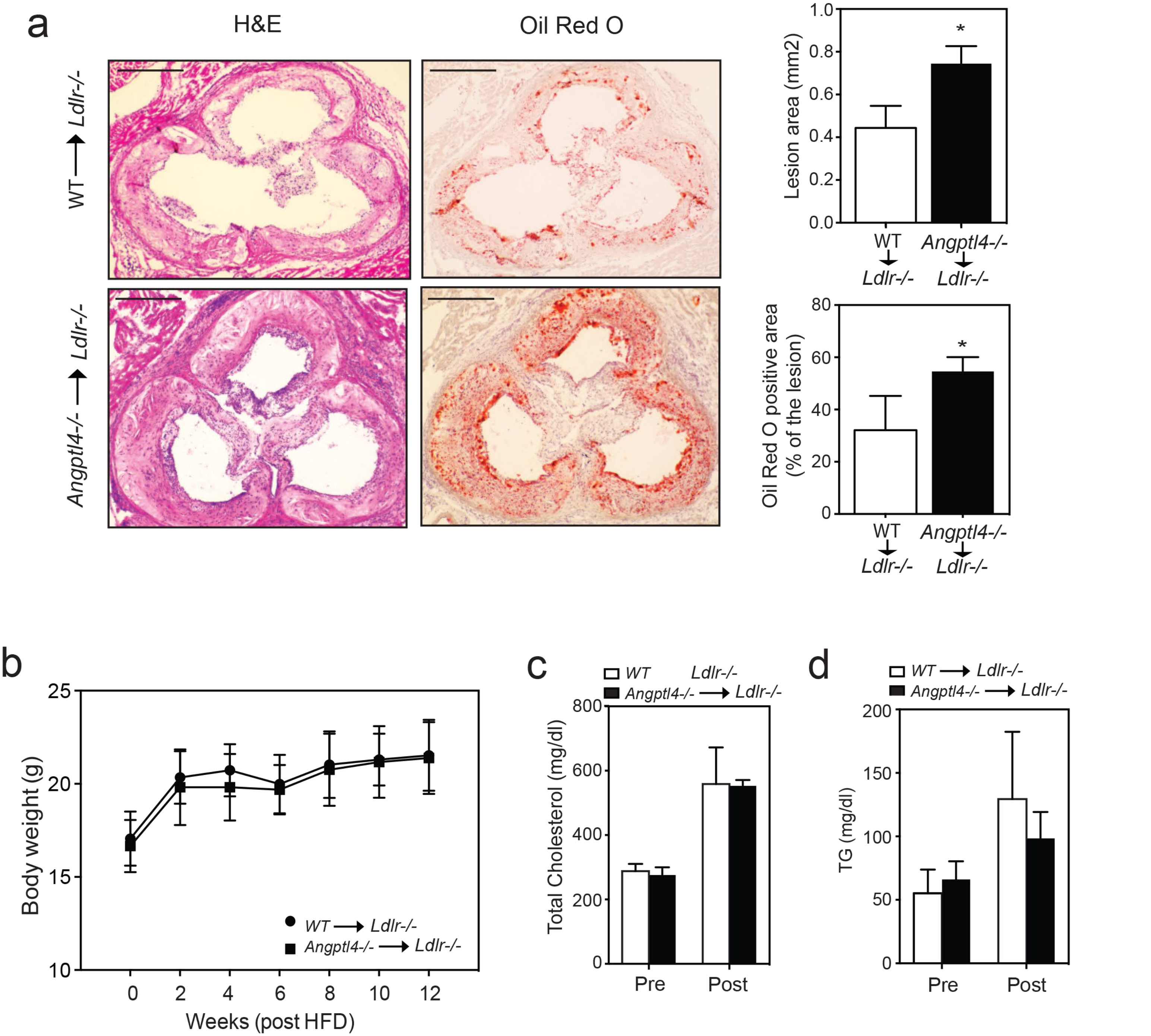

Supplementary Fig. 3. Angptl4$^{-/-}$ bone marrow recipient female Ldlr−/− mice develop bigger atherosclerotic plaques.
(**a**) Representative histological analysis of cross-sections of the aortic sinus from female Ldr chimeras with WT or Angptl4−/− bone marrow (BM) after 12 weeks of WD stained with H&E and Oil Red O. Quantification is shown in the right panel of respective figure (mean ± SEM; n=10 per group; *P<0.05, Student's t-test). Total circulating cholesterol (**b**), plasma triglyceride level (**c**) and body weight (**d**) of female Ldlr−/− chimeras with WT or Angptl4−/− BM before and after 12 weeks on WD (mean ± SEM; n=10 per group). Scale bars = 400 µm.

Supplementary Figure 4

a

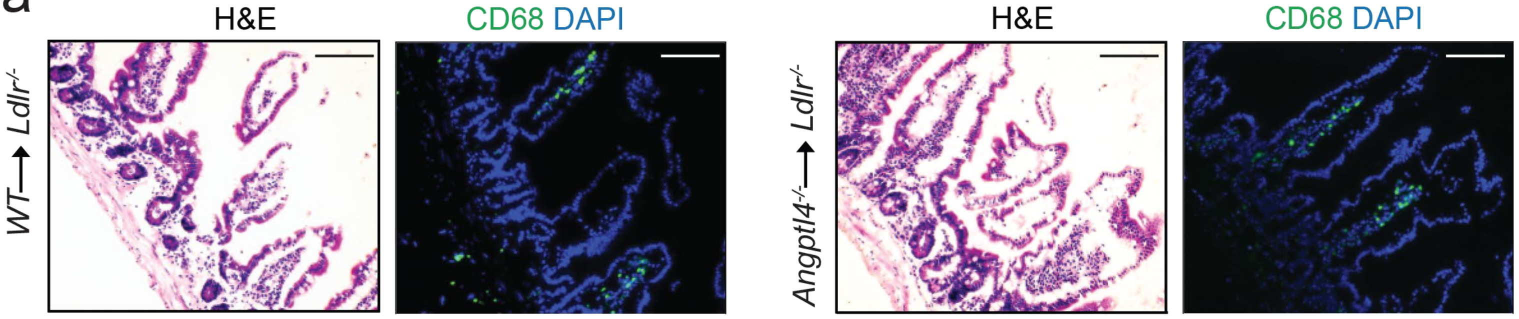

b

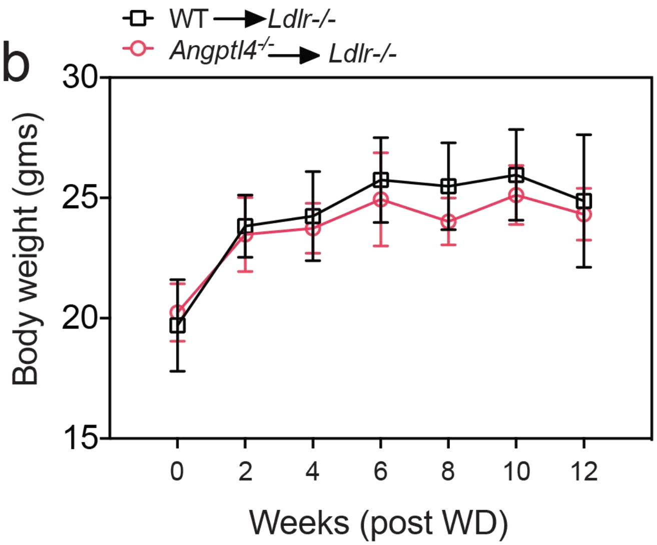

Supplementary Fig. 4. Hematopoietic Angptl4 deficiency does not influence gut inflammation and body weight in Ldlr−/− mice. (**a**) Representative histological analyses of cross-section of the ileum from Ldlr−/− chimeras with WT and Angptl4−/− BM stained with H&E and CD68 after 12 weeks on WD. Scale bars: 400 µm. (**b**) Body weight of male Ldlr−/− chimeras with WT or Angptl4−/− BM before and after 12 weeks on WD (mean ± SEM; n=9 per group).

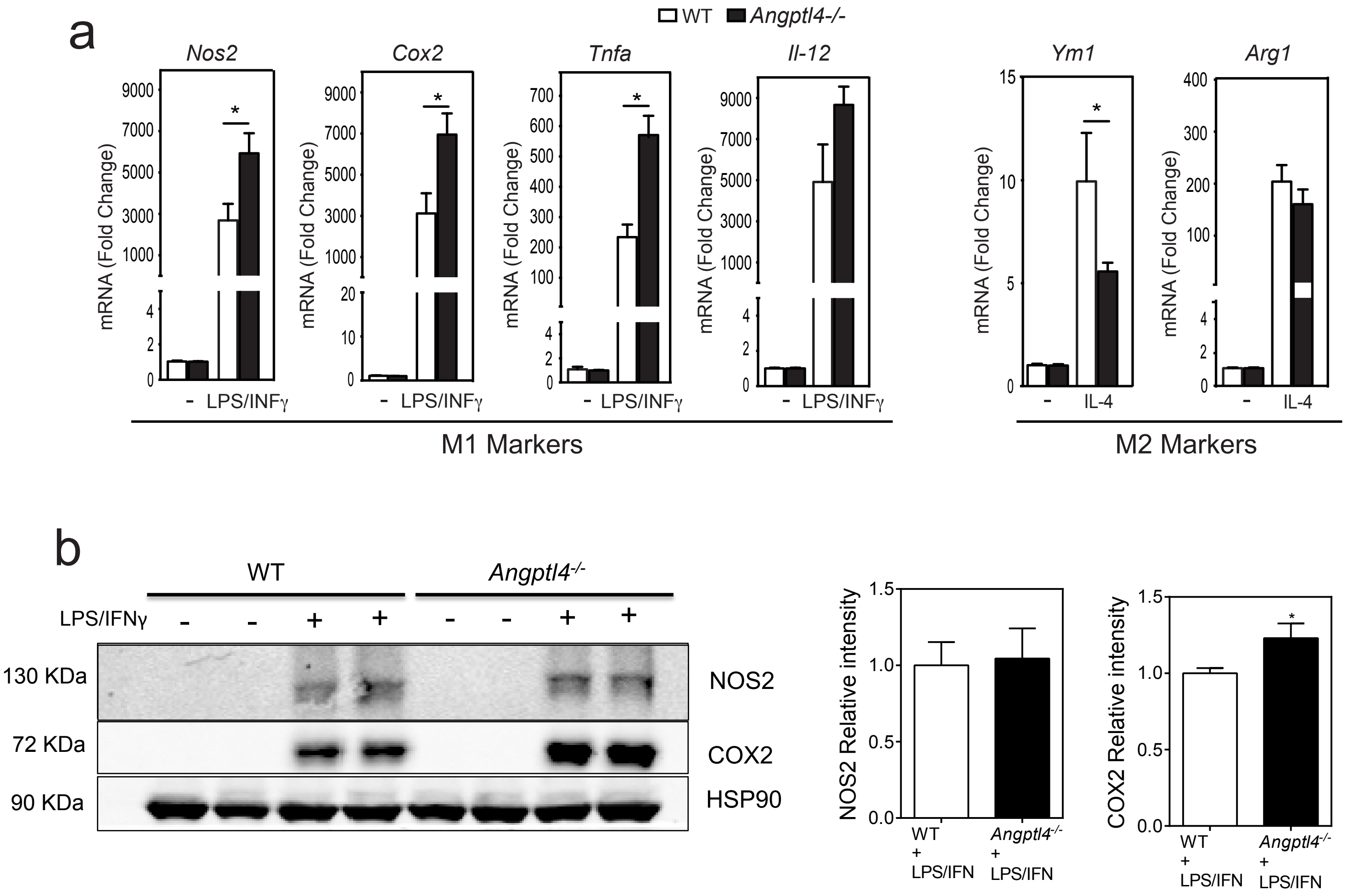

Supplementary Fig. 5. ANGPTL4 deficiency promotes M1 polarization of macrophages. (**a**) mRNA expression of indicated M1 and M2 macrophage polarization genes in bone marrow derived macrophages (BMDM) isolated from WT and Angptl4−/− mice and treated with LPS/IFNg (M1) or IL-4 (M2) for 8 h (mean ± SEM, n>3; *P<0.05, Student's t-test). (**b**) Western blot analysis of indicated proteins from WT and Angptl4−/− BMDMs treated with LPS/IFNg. (**c**) Western blot analysis of indicated proteins from WT and Angptl4−/− BMDMs treated with LPS/IFNg for indicated time points. Quantifications are shown in the lower panels. Full scans of Westerns blots are provided in Supplementary Fig. 9.

Supplementary Figure 5

Supplementary Figure 6

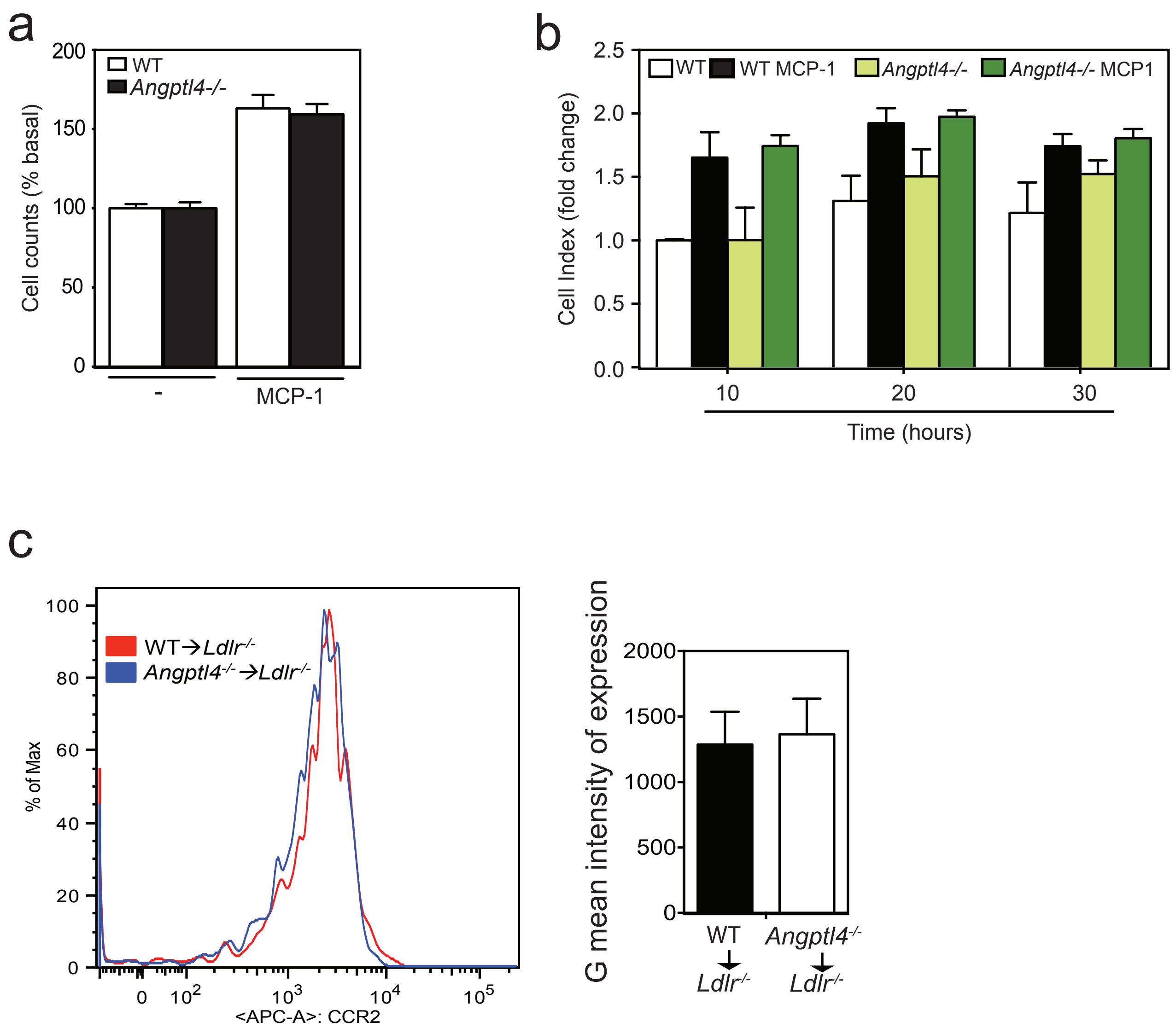

Supplementary Fig. 6. ANGPTL4 deficiency does not influence monocyte migration. (**a** and **b**) Migration of BMDMs from adult WT or Angptl4−/− mice towards MCP-1 (100 ng/ml) using Boyden chamber assay after 16 hours of incubation (mean ± SEM; n=4 in duplicates) (**a**) and Real-time Cell Invasion and Migration (RT-CIM) x CELLigence assay system (mean ± SEM; n=3 in quadruplates) (**b**). (**c**) Representative histogram of the flow cytometry analysis of CCR2 expression in blood monocytes isolated from Ldlr−/− mice transplanted with WT and Angptl4−/− BM and put on WD for 3 months. Quantification of geometric mean intensity from all experiments is on the right panel (mean ± SEM; n=5 per group).

Supplementary Figure 7

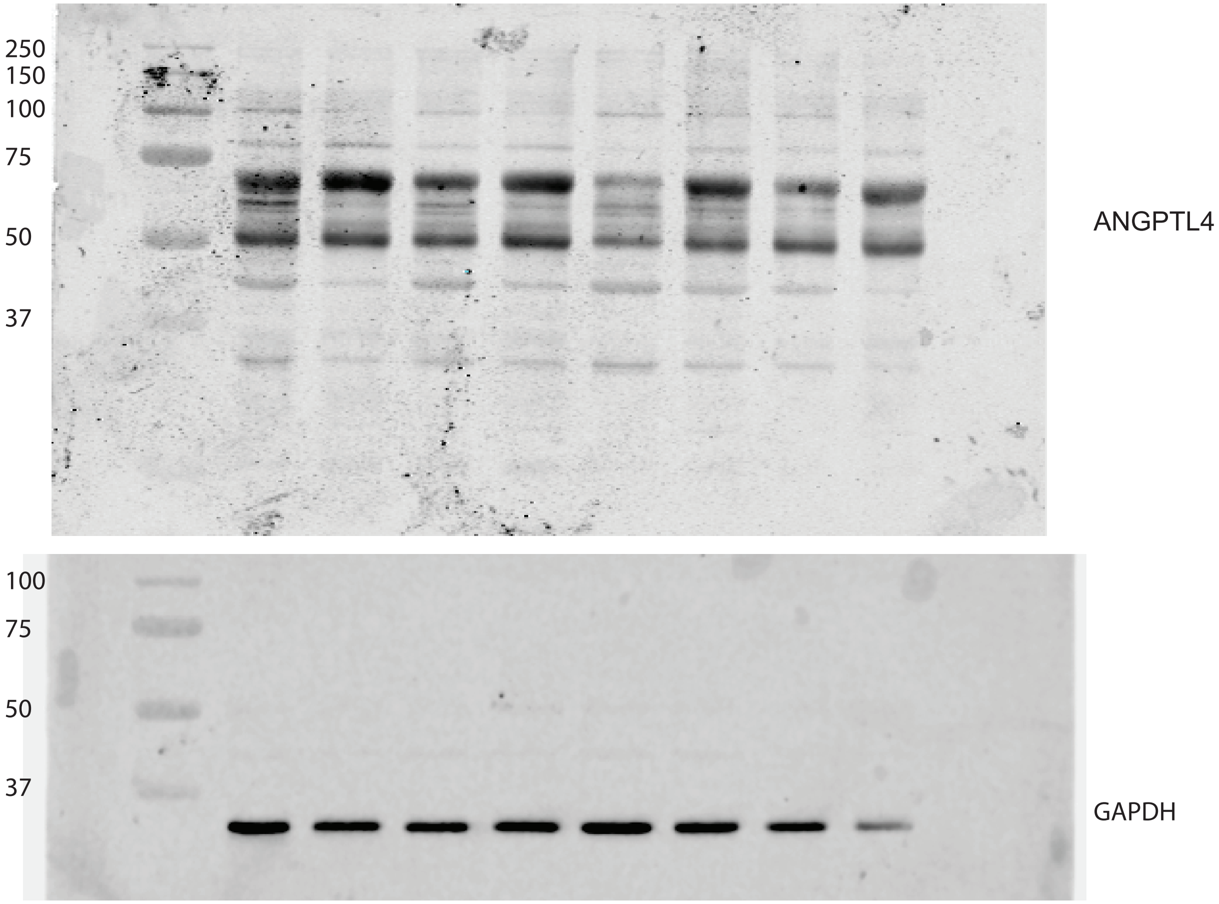

Supplementary Figure 7. Original gel scans for **Fig. 1h**

Supplementary Figure 8

Fig 7f, original western blots

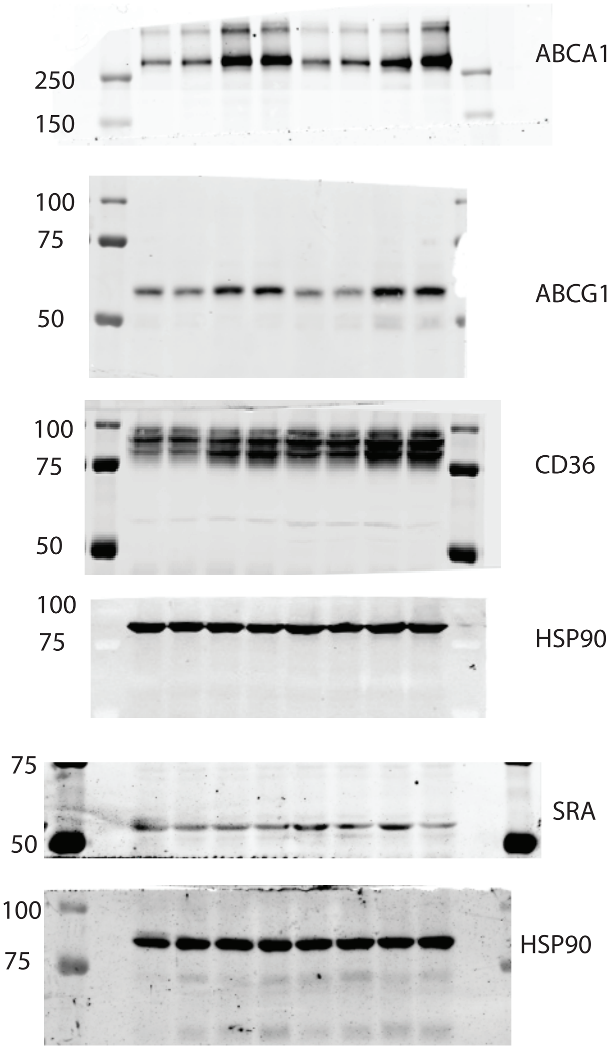

Fig 7g, original western blots

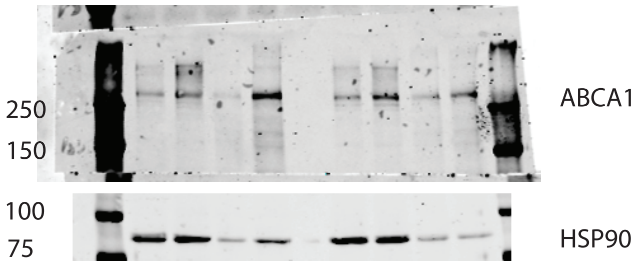

Supplementary Figure 8. Original gel scans for **Fig. 7f** and **Fig 7g**

Supplementary Figure 9

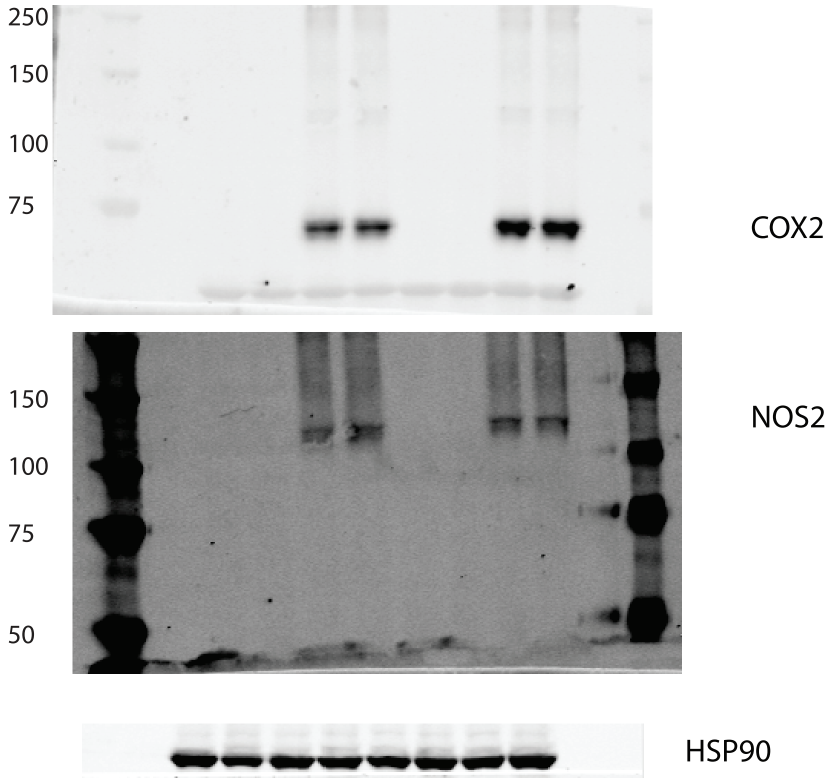

Supplementary Figure 9. Original gel scans for **Supplemental Fig. 5b**

Supplementary Table 1

Mouse primer sequence

**Primer**	**Forward sequence**	**Reverse sequence**
**18S**	5'-ttccgataacgaacgagactct-3'	5'-tggctgaacgccacttgtc-3'
**Angptl4**	5′-caccaatgtttcccccaat-3'	5'-aagataccctttttacgctcctg-3'
**Ear2**	5'-gtggccctcaccgagtatg-3'	5'-ggtctcgatgggtgtcttgc-3'
**F7**	5’-aaaggcgtgccaactcactc-3’	5’-cctacgttctgacatggattcg-3’
**Syn1**	5'-agctcaacaaatcccagtctct-3'	5'-cggatggtctcagctttcac-3'
**Igf1**	5′-gcattgtggatgagtgttgc-3′	5′-ggctcctcctacattctgta-3′
**Lipn**	5'-gggtacattctcgccatcaac-3'	5'-ctcaagccagtaggcattgtc-3'
**Adssl1**	5'-ctcaccttgtgttcgacttcc-3'	5'-agcaaagcccttgagcctttt-3'
**Adam8**	5'-agttcctgtttatgccccaaag-3'	5'-aaaggttggcttgacctgct-3'
**Hyal1**	5'-cctcaaccctgccagtttctc-3'	5'-cccgcttgtcacaccactt-3'
**Acca2**	5'-ctgctacgaggtgtgttcatc-3'	5'-agctctgcatgacattgccc-3'
**Abca1**	5´-ggtttggagatggttatacaatagttgt-3´	5´-cccggaaacgcaagtcc-3´
**Abcg1**	5´-tcacccagttctgcatcctctt-3´	5´-gcagatgtgtcaggaccgagt-3´
**Cd36**	5´-tcctctgacatttgcaggtctatc-3´	5´-aaaggcattggctggaagaa-3´
**Ldlr**	5'-ggtactggcaaccaccattggg-3'	5'-gccaatcgactcacgggttcag-3'
**Nos2**	5´-cagctgggctgtacaaacctt-3´	5´-cattggaagtgaagcgtttcg-3´
**Glut1**	5'-cagttcggctataacactggtg-3'	5’-gcccccgacagagaagatg-3’
**Cox-2**	5’-acctctccaccaatgacgtg-3’	5’- gggagagagttcatccctga-3’
**Tnfα**	5´-ccctcacactcagatcatcttct-3´	5´-gctacgacgtgggctacag-3´
**Il-12b**	5’- ggaagcacggcagcagaata-3’	5’- aacttgagggagaagtaggaatgg-3’
**Ym1**	5'-gcagaagctctccagaagcaatcctg-3'	5'-attggcctgtccttagcccaactg-3'
**Arg1**	5´-ttgggtggatgctcacactg-3´	5´-ttgcccatgcagattccc-3´
**Mcp-1**	5’- actcattcaccagcaagatg-3’	5’- tgtaggttctgatctcatttgg-3’
**Cd68**	5'-ccaattcagggtggaagaaa-3'	5'-ctcgggctctgatgtaggtc-3'
**Hmgcr**	5-cttgtggaatgccttgtgattg-3'	5'-agccgaagcagcacatgat-3'
**Il-6**	5´-agttgccttcttgggactga-3´	5´-tccacgatttcccagagaac-3´
**Ccl3**	5’-ttctctgtaccatgacactctgc-3’	5’-cgtggaatcttccggctgtag-3’
**Icam-1**	5'-ctcggagacattagagaacaatgc-3	5'-gggaccacggagccaatt-3'
**Vcam-1**	5'-gcgtttagtgggctgtctatctg-3'	5'-ctgaatacaaaacgatcgctcaa-3'
**Sele**	5'-agctacccatggaacacgac-3'	5'-cgcaagttctccagctgtt-3'

Human primer sequence

**ANGPTL4**	5'-ctccacttgggaccaggatca-3'	5'-atggctgcaggtgccaaac-3'
**ABCA1**	5'-ggtgatgtttctgaccaatgtga-3'	5'-tgtcctcataccagttgagagac-3'
**HMGCR**	5´-gtcattccagccaaggttgt-3´	5´-gggaccacttgcttccatta -3´

Genotyping primers

**Angptl4**	P1	5'-gtgcaaccgtgaaacgctta-3'
	P2	5'-agccagcaagttcatctcgt-3'
	P3	5'-gacagtatcggcctcaggaa-3'
**Ldlr**	P1	5'-gcgatggatacactcactgc-3'
	P2	5'-aatccatcttgttcaatggccgatc-3'
	P3	5'-ccatatgcatccccagtctt-3'

Supplementary Table 1. PCR primer sequences
